# The Laminin Response in Inflammatory Bowel Disease: Protection or Malignancy?

**DOI:** 10.1371/journal.pone.0111336

**Published:** 2014-10-27

**Authors:** Caroline Spenlé, Olivier Lefebvre, Joël Lacroute, Agnès Méchine-Neuville, Frédérick Barreau, Hervé M. Blottière, Bernard Duclos, Christiane Arnold, Thomas Hussenet, Joseph Hemmerlé, Donald Gullberg, Michèle Kedinger, Lydia Sorokin, Gertraud Orend, Patricia Simon-Assmann

**Affiliations:** 1 Inserm U1109, MNT3 team, Strasbourg, France; 2 Université de Strasbourg, Strasbourg, France; 3 LabEx Medalis, Université de Strasbourg, Strasbourg, France; 4 Fédération de Médecine Translationnelle de Strasbourg (FMTS), Strasbourg, France; 5 Department of Gastroenterology, CHRU Hautepierre, Strasbourg, France; 6 Department of Anatomy and Pathology, CHRU Hautepierre, Strasbourg, France; 7 Inserm U843, Paris, France; 8 INRA, UMR1319, Jouy-en-Josas, France; 9 AgroParisTech, UMR Micalis, Jouy-en-Josas, France; 10 Inserm U1121, Strasbourg, France; 11 Department of Biomedicine, University of Bergen, Bergen, Norway; 12 Institute of Physiological Chemistry and Pathobiochemistry, University of Münster, Münster, Germany; Cedars-Sinai Medical Center; UCLA School of Medicine, United States of America

## Abstract

Laminins (LM), basement membrane molecules and mediators of epithelial-stromal communication, are crucial in tissue homeostasis. Inflammatory Bowel Diseases (IBD) are multifactorial pathologies where the microenvironment and in particular LM play an important yet poorly understood role in tissue maintenance, and in cancer progression which represents an inherent risk of IBD. Here we showed first that in human IBD colonic samples and in murine colitis the LMα1 and LMα5 chains are specifically and ectopically overexpressed with a concomitant nuclear p53 accumulation. Linked to this observation, we provided a mechanism showing that p53 induces LMα1 expression at the promoter level by ChIP analysis and this was confirmed by knockdown in cell transfection experiments. To mimic the human disease, we induced colitis and colitis-associated cancer by chemical treatment (DSS) combined or not with a carcinogen (AOM) in transgenic mice overexpressing LMα1 or LMα5 specifically in the intestine. We demonstrated that high LMα1 or LMα5 expression decreased susceptibility towards experimentally DSS-induced colon inflammation as assessed by histological scoring and decrease of pro-inflammatory cytokines. Yet in a pro-oncogenic context, we showed that LM would favor tumorigenesis as revealed by enhanced tumor lesion formation in both LM transgenic mice. Altogether, our results showed that nuclear p53 and associated overexpression of LMα1 and LMα5 protect tissue from inflammation. But in a mutation setting, the same LM molecules favor progression of IBD into colitis-associated cancer. Our transgenic mice represent attractive new models to acquire knowledge about the paradoxical effect of LM that mediate either tissue reparation or cancer according to the microenvironment. In the early phases of IBD, reinforcing basement membrane stability/organization could be a promising therapeutic approach.

## Introduction

Inflammatory bowel diseases (IBD) that comprise Crohn’s disease (CD) and ulcerative colitis (UC) are multifactorial pathologies where genetic and environmental factors initiate and drive the pathology [1]. Chronic inflammation results from a homeostatic imbalance, a phenomenon that also characterizes tumor development [2]. IBD are characterized by various degrees of inflammation of the intestine causing epithelial damage, among others [3]. In general, the intestinal epithelium is able to repair itself by the restitution of the epithelial layer. In response to chronic ulceration, Ulcer Associated Cell Lineage glands (UACL; [4]) expressing particular trefoil factor (TFF) and mucin molecules [5]–[7] are found that appear to promote mucosal repair and healing. Both forms of IBD, CD and UC, have an inherent risk of progression into cancer with a similar occurrence in patients with colonic CD to that with UC to develop colitis-associated cancer [8], [9]. Repeated tissue destruction and repair together with oxidative damage can trigger mutagenesis and may serve as cancer initiating events. In this process, a possible causative role for mutated p53 tumor-suppressor gene is more and more evident. Indeed, point mutations often resulting in a p53 gain of function, have been identified in neoplastic progression of UC [10]–[12] and were shown to promote inflammation induced progression into intestinal cancer [13].

Inflammatory responses are often associated with remodeling of the extracellular matrix (ECM) as evidenced in wound healing and tissue repair. Profound alterations in ECM expression and ECM binding integrin adhesion receptors have been found in a number of inflamed tissues [14], [15]. The intestinal basement membrane (BM) represents a specialized ECM network that separates epithelial cells from the underlying connective tissue and is mainly composed of collagen IV, laminins (LM), perlecan and nidogens. The BM functions as a physical and chemical barrier. Several human disorders result from or are associated with defects in BM assembly or composition [16]. Two susceptibility loci linked to ECM candidates, *ECM1* and *LAMB1*, were found associated with UC [17]. LM are a family of BM glycoproteins, each containing an α-, β- and γ- chain that assemble into characteristic heterotrimers. LM, and in particular their α chains carrying the cell binding domains, have been shown to be important for cell adhesion, migration and proliferation; they are also known to protect cells from apoptosis [16], [18], [19]. Immunodetection has revealed that several LM isoforms exist in the human intestine, including LM-111 (α1β1γ1), LM-511 (α5β1γ1) and LM-332 (α3β3γ2) which show developmental and position specific expression along the crypt-villus axis [20], [21]. In IBD, inflammation leads to mucosal ulceration and subsequent tissue repair that implies a continuous remodeling of the BM. Altogether, LM may play an yet unknown instrumental role in the inflammation response.

Limited data exist on the expression of epithelial BM constituents in IBD. Altered immunoreactivity of BM constituents has been described in IBD with an increase in LMα3 and LMα5 chains in the crypt region of inflamed segments of CD small intestine in particular [22], [23]. Among the genes identified by mRNA profiling in inflamed UC colon, those associated with tissue remodeling such as *LAMA2* (encoding LMα2 chain) have been reported to be overexpressed [24]. *In vitro* studies using normal intestinal epithelial cells demonstrated that the two inflammatory cytokines TNF-α and IFN-γ synergistically modulate the expression and secretion of LMα5 and LMγ2 chains [25]. Although sporadic, these data strengthen the notion that the balance of different LM isoforms is crucial for tissue homeostasis and imply that LM contribute to the inflammation response [26].

As the BM is an important actor of the intestinal barrier, we addressed in the present study the role of LM in IBD. First, we defined the expression of the major LM chains in colon specimens from IBD patients and from a murine colitis model. By immunofluorescence we showed a high expression of LMα1 and LMα5 in the inflamed tissue that was associated with nuclear p53. We addressed a potential role of p53 in inflammation-induced LM expression and observed induction of *LAMA1* in a p53 dependent manner. We addressed the potential role of elevated LM expression in IBD by inducing colitis in transgenic mice that overexpressed LMα1 or LMα5 and demonstrated a protective effect of these molecules against inflammation. However, in context of carcinogenic mutations, high LMα1 or LMα5 levels enhanced progression of chemically-induced colitis into cancer.

## Materials and Methods

### Human specimens and scoring of inflammation

Paraffin embedded tissue was obtained from 25 patients who had undergone intestinal resection for acute CD of the colon and from 7 patients with UC. As controls, non-inflammatory colon samples for each patient as well as 15 colon samples distant from colon carcinoma were analyzed. In parallel, adjacent samples for immunofluorescence staining were embedded in Tissue-Tek (Sakura, Labonord), immediately frozen on dry ice and kept at −80°C until later use. The inflammatory state was assessed by a pathologist using the Riley score [27] and confirmed by immunostaining for CD45, TLR4 (**Figure S1**) and for CD68 (not shown). IBD samples were obtained with the written informed consent of patients prior to inclusion in the study. The Institutional Review Board of the “Centre de Ressources Biologiques” (Association française de normalisation: 2010/39043.2) of the Hautepierre hospital (Strasbourg, France) has approved the study on human samples.

### Mouse models, genotyping and animal experiments

Methods about the generation of Tg-*lama1* and Tg-*lama5* mice, induction of colitis and of cancer-associated colitis as well as cytokine measurements are provided in the **Methods S1**section. All procedures with animals were performed under a protocol approved by the “Direction Départementale de la Protection des Populations” (agreement number: 67–261) and in accordance with the ethical rules for the care and use of animals for research (Comité d’éthique pour l’experimentation animale, CEEA35 and “Institut national de la santé et de la recherche médicale” E67-482-21 for the agreement of the animal house); all efforts were made to minimize suffering.

### Expression analysis at tissue and RNA levels

Antibodies used and primer sequences are listed in **Table S1** and **Table S2**, respectively. Details concerning immunohistochemistry, immunofluorescence, histology, assessment of apoptosis, RNA extraction and RT-qPCR are provided in the **Methods S1**section.

### *In vitro* analysis of LM impact on p53 expression and on NF-κB activity

HCT116 human colon carcinoma cells were plated onto 6-well uncoated plates or plates coated with LM-111 or Caco-2 derived LM-511 as described previously [28], and RNA was extracted 48 h, 72 h or 96 h after plating. RT-qPCR for p53 mRNA was then performed with primers listed in **Table S2**. For Western blot analysis, antibodies to p53 and to actin (internal control) are listed in **Table S1**. To address a possible impact of LM on NF-κB signaling, stable NF-κB reporting HT-29 cells [29] were seeded onto 6-well control plates or onto plates coated with LM-111, Caco-2 derived LM-511, human tenascin-C [30] or rat tail collagen I at 5 µg/cm^2^. Luciferase activity was determined using the Luciferase Assay System (Promega, France) according to the manufacturer’s instructions. Details about cell culture conditions are described in the **Methods S1**section.

### AFM measurements

HT29 epithelial cells expressing or not the LMα1 chain (H11 and B8T clones respectively [31]) were cultured for ten days. Cell-derived matrices deposited on the cell culture dishes were isolated following removal of the cells after a combined treatment of 1% Triton X-100 with 10 mM EDTA [32]. AFM experiments were realized by using a Bioscope Catalyst apparatus (Bruker Nano Surfaces Division, Santa Barbara, CA USA). The silicon nitride cantilever with a spring constant of 0.06 N.m^−1^ and a 5 µm radius borosilicate particle attached to the tip (Novascan Technologies, Ames, IA USA) was navigated over the cell-derived matrix with approach/withdraw velocities of 20.6 µm.s-1. Controlled deformations were applied to the sample and the compressive feedback forces were measured through cantilever deflection. On every culture dish, 25 areas were probed in 3 different sample regions of 50×50 µm each. The Young’s-modulus (E) of the probed material was calculated by fitting the contact part of the measured approach force curves to a standard Hertz model for a spherical indenter (tip) of radius *R*. Elasticity (E) of the probed material will reflect the mechanical properties of the ECM deposited by the cells.

### Transfection and infection with p53 or sh-*TP53*

For transfection experiments, HCT116 cells were grown onto 6-well plates (500 000 cells per well) and transfected with 3 µg of either pCMV-Neo-Bam p53 wt, pCMV-Neo-Bam p53 V143A, pCMV-Neo-Bam p53 R248W, pCMV-Neo-Bam p53 R249 S, pCMV-Neo-Bam p53 R175 H, pCMV-Neo-Bam p53 R273 H, or pCMV-Neo-Bam (Addgene, Cambridge, MA) using JetPEI™ reagent (PolyPlus Transfection, Illkirch, France) according to the manufacturer’s instructions. RNA was extracted at different time intervals (6 h, 48 h, 3 days or 4 days) after transfection. For the 4 days time point of analysis, cells were re-transfected at day 3.

Five different MISSION^R^ lentiviral shRNA clones for human *TP53* and a non-target shRNA control lentivirus (Sigma-Aldrich, St Louis, MO) were tested in a first round in HCT116 cells. Populations of lentiviral HCT116 infected cells were selected using 1 µg/ml puromycin (Invitrogen, France). Efficiency of *TP53* inhibition was determined by RT-qPCR. Two stable HCT116 sh-*TP53*(1) and sh-*TP53*(2) cell lines showing an inhibition of p53 expression of 86% and 92% respectively were selected for further experiments. Cells were treated with irinotecan (20 µM, 48 h; Roche Diagnostics, Meylan, France) and RNA extracted for determination of *LAMA1* transcripts by RT-qPCR.

### Chromatin Immunoprecipitation Assay (ChIP)

The ChIP Assay was performed as recommended by the manufacturer (EZ-Magna ChIP™ G kit, Millipore, France). HCT116 cells were transfected by either pCMV-Neo-Bam or pCMV-Neo-Bam-p53-wt 48 h before the experiment. Immunoprecipitation was performed using two different anti-TP53 antibodies (**Table S1**) or normal mouse IgG as negative control. Immunoprecipitated DNA was then used as a template for PCR. Putative p53 binding sites on a 7 kb sequence of the *LAMA1* promoter and on the first 5 kb of intron-1 were located using MatInspector Professional program (www.genomatix.de). Primers surrounding each p53 binding site are detailed in **Table S2**.

### Statistical analysis

When data followed a Gaussian distribution, statistical analysis was performed using the one sample t-test (*TP53* gene transfection), the t-test (AFM) or the Anova’s test with Tukey’s multiple comparison test (NF-κB reporter assay). Otherwise, the Mann Whitney test (ELISA assay and irinotecan data) was used to verify significance of the observed differences. All statistical analyses was performed using the GraphPad software.

## Results

### Concomitant high expression of LMα1 and LMα5 correlates with nuclear p53 in colitis

**Figure 1:**
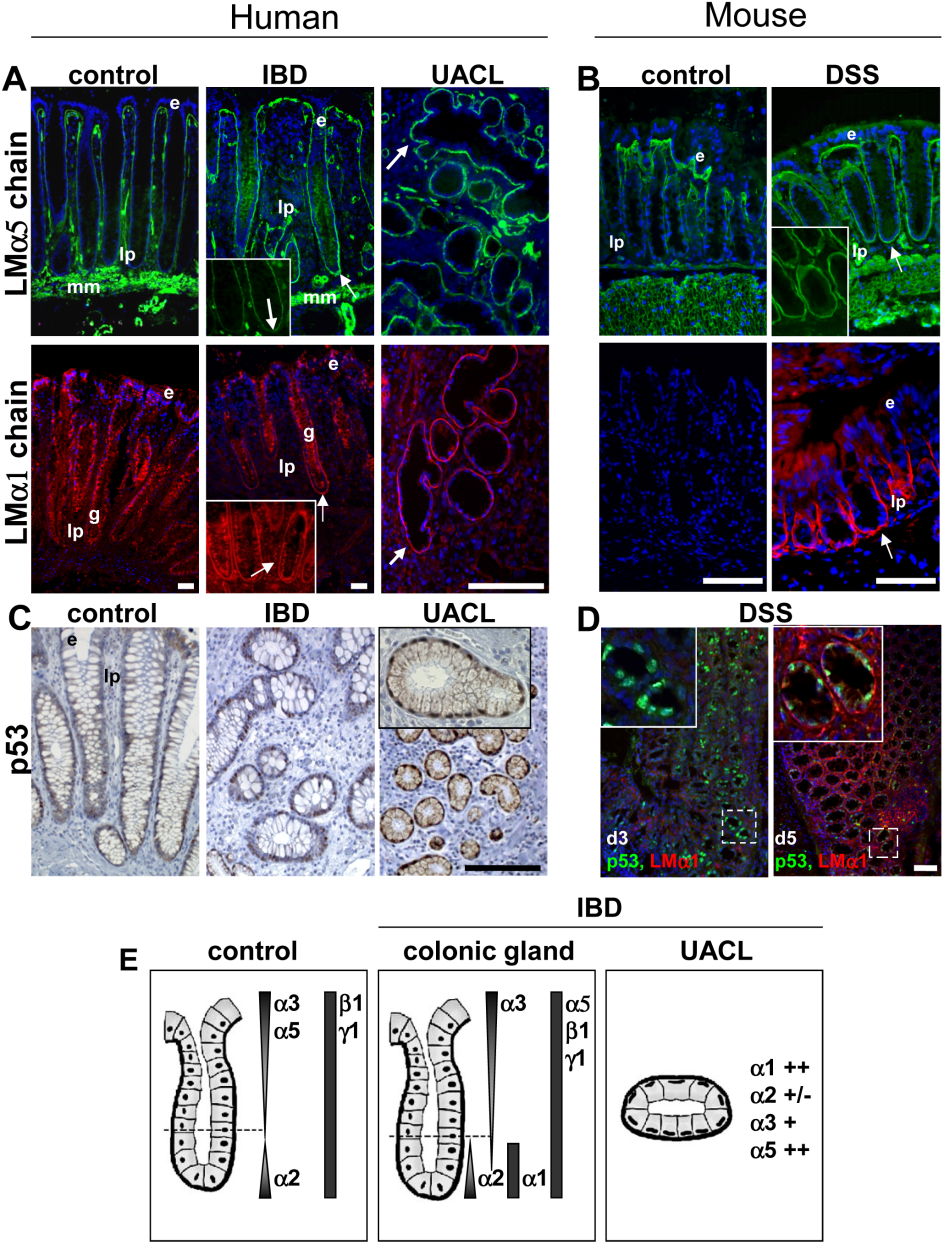
Inflammation response triggers expression of LMα1/α5 and nuclear p53 accumulation in human and murine colitis. (**A**) Expression of the LMα5 and LMα1 chains in normal mucosa (control), in mild inflamed glands and around UACL from colon of patients with IBD. Immunostaining for LMα5 in IBD samples is extended along the colonic glands with a strong staining at the deeper crypt region and is highly expressed in UACL (arrows). LMα1 is detected selectively at the BM at the crypt bottom in IBD samples and in UACL while it is absent from uninflamed regions; unspecific cytoplasmic immunoreactivity is seen in goblet cells (g). Inset: higher magnification of the deeper crypt region. (**B**) Expression of LMα5 and LMα1 in cryosections of colon from control and DSS-treated mice. Note, as in human IBD, high expression of LMα5 and LMα1 was observed in mouse colitis at the bottom of the colonic glands. (**C**) Nuclei of epithelial cells from inflamed colonic segments and in UACL of IBD patients were positive for p53 while nuclei of the adjacent normal crypts showed rare p53-positive cells scattered within the glands. (**D**) Expression of p53 (green) and LMα1 (red) in colon samples from DSS-treated mice. At day 3 after treatment, p53 immunoreactivity was present in numerous nuclei within epithelial cells while LMα1 co-staining was weak; inset: enlarged area with nuclear p53 expression. At day 5, intense LMα1 staining was observed surrounding weaker p53-positive glands (inset). (**E**) The diagram summarizes the distribution of the main LM chains found in normal colonic mucosa (control), in mild inflamed glands and around UACL from colon of patients with IBD. Note that only the staining at the epithelial BM is schematically represented for clarity. e: epithelial cells; lp: *lamina propria*; mm: *muscularis mucosae*; arrows: ectopic staining at crypt bottom and staining around UACL. Scale bars: 50 µm (human), 25 µm (mouse).

A comprehensive analysis of LM distribution was conducted on IBD human colon samples with chain specific LM antibodies. Since in advanced stages of ulceration a detachment of the epithelium together with the BM was observed, only specimens with a mild inflammation could be investigated. Significant differences in the spatial distribution of LMα1 and LMα5 chains were detected at the BM level in all IBD tissues (CD and UC) compared to control segments. While in normal colon mucosa, the LMα5 chain presented a gradient of expression along the colonic crypts and was absent at the bottom, a significant up-regulation was obvious in the IBD samples (**Figure 1A**). This resembles an expression pattern that had been described for the inflamed proximal small intestine [22]. Concomitantly, there was an induction in the crypt region of the LMα1 chain that is usually absent from the BM in the normal adult colon (**Figure 1A**). Distribution of LMα2, LMα4, LMβ1, LMβ2, LMγ1 chains along the BM region of colon glands was similar between IBD and control tissues while the gradient of LMα3 and LMγ2 was slightly extended in IBD (**Figure S2** and data not shown). Thus fine examination by immunodetection allows us to show that LMα1 and LMα5 chains are ectopically and concomitantly expressed at the bottom of the colonic crypts of IBD patients.

In CD and UC colon samples we also found the UACL glands which are believed to play a role in tissue regeneration [4]. These glands can be easily visualized by a stronger staining with PAS, their typical appearance (epithelial cells with flat nuclei aligned along the basal pole of the cells) and their particular expression pattern of gastrointestinal mucins and trefoil peptides [4], [6] (**Figure S3**). We further characterized the molecular composition of these UACL by using several markers (**Figure S4**, and data summarized in **Table S3**). We found that UACL still expressed epithelial characteristics, were positive for repair proteins and for actors of the Wnt-signaling pathway. Interestingly, LMα1 and LMα5 were strongly expressed around the UACL glands (**Figure 1A**). This was different to LMα2 and LMα3 which were irregularly and weakly expressed (**Figure S2**). Altogether, these data, summarized in **Figure 1E**, suggest a functional role of the LMα1 and LMα5 chains in IBD.

To experimentally mimic the inflammation phase of human IBD, we induced DSS-driven colitis in mice. These mice presented obvious signs of distal colonic inflammation that were identified along the Swiss-roll of the colon (**Figure S5**). Within the inflamed regions, areas with ectopic expression of LMα5 were observed at the bottom of the crypts concomitant to a striking induction of LMα1 (**Figure 1B**). These data corroborate our results in human IBD, suggesting that the murine model phenocopies important features of the human disease.

Integrins are the main cellular receptors known to bind the LMα chains, in particular integrins α6β1 and α6β4 [19]. To determine whether expression of these integrins is potentially altered in IBD and murine colitis, we determined their expression by immunofluorescence tissue staining. Whereas integrin β1 expression was not altered in the inflamed tissue (data not shown), α6β4 integrin was strongly expressed at the bottom of the colonic crypts from mouse-colitis and IBD tissues as well as in the UACL. This strong α6β4 integrin staining, located at the same place where LMα1 and LMα5 are overexpressed, suggests that cells may interact with both LM through this integrin (**Figure S6**).

During the ulceration process, cellular stress arises that typically triggers a p53 response in order to guarantee genome integrity [33]. Therefore we investigated the expression and location of p53 in human IBD and in murine colitis by immunohistochemistry using antibodies that detect both wild-type and mutated p53 [34]. Histologically normal epithelium showed only sporadic nuclear staining for p53. In contrast, most of the nuclei from UACL were strongly positive for p53 and often neighboring glands presented also some nuclear p53 expression (**Figure 1C**). Similar to the human IBD tissue, p53 was also strongly expressed in nuclei of the murine inflamed colon. This is particularly obvious at early stages of inflammation (3 days after DSS treatment). At a later time point, 5 days after DSS treatment, the number of p53-positive cells decreased concomitantly with an increased LMα1 expression **(** **Figure 1** **D**).These data are consistent with a potential role of p53 in regulating *LAMA1* transcription upon inflammation.

### *LAMA1* expression is triggered by p53

**Figure 2:**
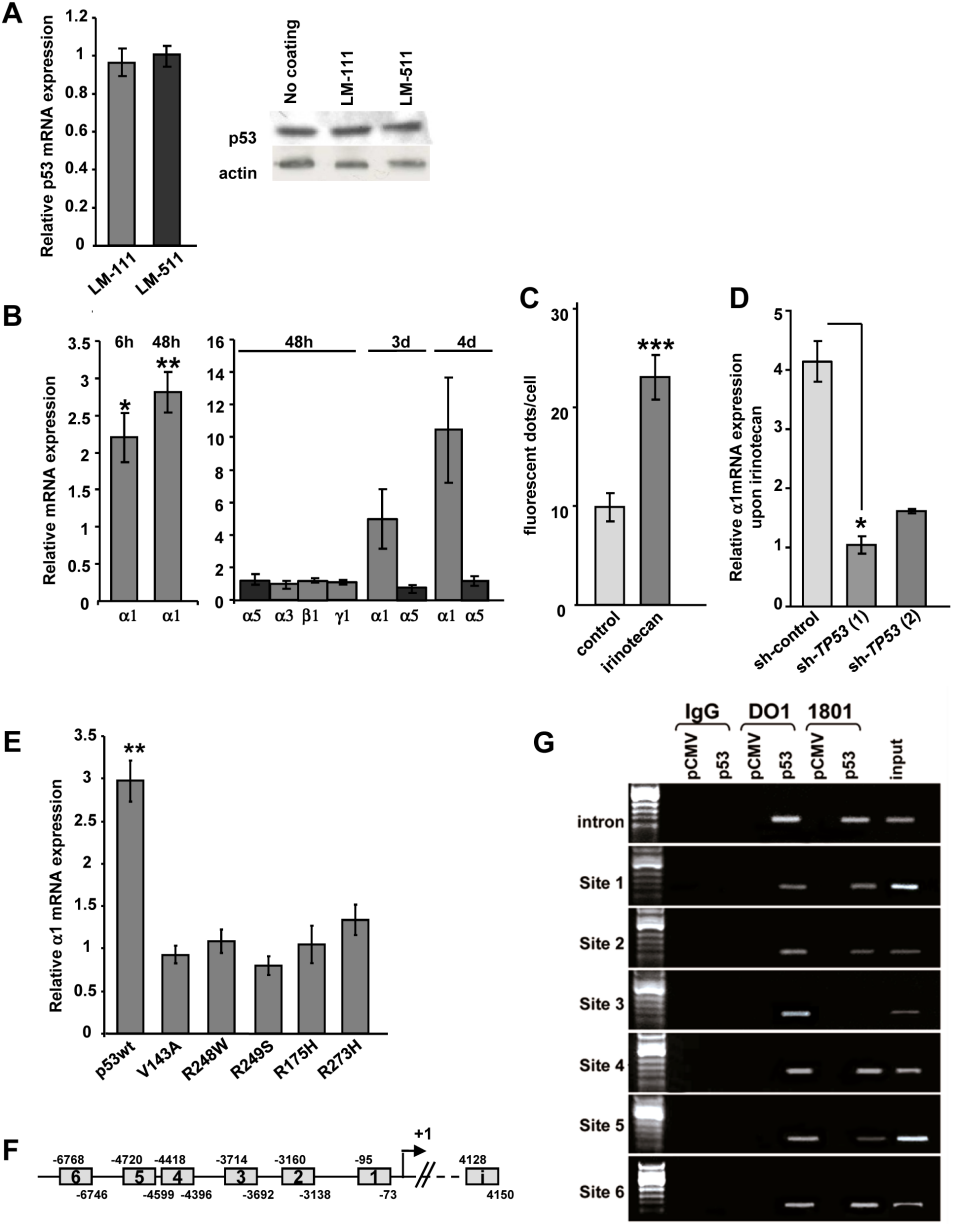
Wild-type p53 induces LMα1 expression in epithelial cells via binding to the *LAMA1* promoter. (**A**) HCT116 cells were seeded on LM-111 or LM-511-enriched matrix. (Left) Endogenous p53 mRNA was quantified by RT-qPCR (normalized to *GAPDH*) and expressed as ratio relative to the control (plastic dishes). The values are given as a mean +/− SEM of 9 independent experiments which were pooled (n = 3 at 2 days; n = 3 at 3 days; n = 3 at 4 days). (Right) Representative immunoblot of p53 and actin from HCT116 cells in the different conditions. Data show that LM substrata do not activate endogenous expression of p53. (**B**) Relative mRNA expression of LM chains in HCT116 cells upon transfection with a *TP53* vector assessed at different time points. Transcript levels were determined by RT-qPCR and normalized to *GAPDH* and are presented relative to the control vector (n = 3 to 4 experiments, except for α1 at 6 h n = 6 and at 48 h n = 8). Data show that wild-type (wt) p53 induces selectively and progressively LMα1 mRNA levels. (**C**) Semiquantitative analysis of intracellular LMα1 after immunofluorescence staining of HCT116 cells (48 h of irinotecan treatment). Note a 2.3-fold intracellular deposition of LMα1 in irinotecan-treated cells as compared to untreated cells (n = 15). (**D**) Expression of LMα1 mRNA from two independent stable sh-*TP53* HCT116 cell lines and in a sh-RNA control cell line, upon treatment with irinotecan. After 48 h, relative mRNA expression of LMα1 was assessed by RT-qPCR and normalized to *GAPDH;* values are given as ratios relative to those found in the corresponding untreated cells (n = 3). In p53-deprived cells, irinotecan was unable to stimulate LMα1 expression. (**E**) Relative *LAMA1* mRNA expression in HCT116 cells following transfection with wt or mutants p53 (ratios calculated as stated above; n = 6). LMα1 mRNA were only activated by wt p53. (**F**) Diagram showing the location of putative p53 binding motifs (sites 1 to 6) in the *LAMA1* promoter 7 kb upstream of the transcription site and in the 5 kb region of intron 1 (i). (**G**) Chromatin immunoprecipitation experiments. Chromatin was prepared from HCT116 cells transfected with either the control (pCMV) or *TP53* vector (p53). Cross-linked p53-DNA complexes were immunoprecipitated by either IgG (negative control) or anti-p53 antibodies (DO1 or 1801) followed by PCR amplification using primers that flank the putative p53 binding sites. Input represents chromatin before immunoprecipitation. Note that p53 binds to 7 candidate p53 binding sites. Bars represent mean +/−SEM; *p<0.05, **p<0.01, ***p<0.001.

First to exclude a potential regulation of p53 by LMα1 or by LMα5, we cultured human colorectal HCT116 cells on LM-111 or LM-511-enriched matrices and p53 expression was determined by RT-qPCR and immunoblotting. We noticed that neither of the LM substrata had an impact on p53 mRNA and protein levels (**Figure 2A**). To address the hypothesis of p53-dependent regulation of LM, we performed transfection experiments. More precisely, HCT116 cells were transfected with a wild type *TP53* expression plasmid to examine whether such an ectopic expression would result in increased expression of endogenous laminin mRNA. **Figure 2B** shows that expression of *LAMA1* mRNA in HCT116 cells was increased 2.2-fold as early as 6 h and further increased by 2.8- fold at 48 h after transfection with the p53 plasmid. Our study revealed also a time-dependent increase of *LAMA1* mRNA up to 4 days. In contrast to LMα1, mRNA levels for LMα3, LMα5, LMβ1 and LMγ1 were not modified upon p53 overexpression (**Figure 2B**).

To address the role of endogenous p53 on LMα1 induction, the topoisomerase I inhibitor irinotecan was used to trigger p53 expression [35] in HCT116 cells. Semi-quantitative immunodetection of the LMα1 protein in irinotecan-treated cells revealed a 2.3-fold increase of the p53 protein (**Figure 2C**). To further confirm the p53-dependent *LAMA1* induction, we determined *LAMA1* mRNA levels upon a knockdown of p53 by shRNA-technology. We derived two stable HCT116 sh-*TP53* cell lines where the inhibition of p53 expression reached up to 92% (see Materials and Methods) as compared to sh-control infected cells. In these p53-deprived cells, irinotecan did not induce *LAMA1* mRNA expression (**Figure 2D**).

As p53 mutants can be gain-of-function [36] we investigated whether common human colorectal cancer-derived p53 mutants ([37] and http://p53.iarc.fr) also induced *LAMA1* transcription. Therefore, we expressed 5 different point mutants of p53 upon transfection in HCT116 cells and determined *LAMA1* mRNA levels. We noticed that only wild-type but none of the tested p53 mutants induced *LAMA1* expression (**Figure 2E**).

To address whether p53 induces *LAMA1* transcription by promoter binding, we searched for potential p53 binding sites in the 3′ upstream regulatory sequence of p53. By sequence analysis, we identified seven putative p53 binding sites in the 7 kb *LAMA1* sequence of the promoter and in the first 5 kb of intron-1 (**Figure 2F**). We used a ChIP assay to address whether p53 can bind to the *LAMA1* regulatory sequences. By using two different p53 antibodies we found that these putative p53 binding sites were amplified in the immunoprecipitates (**Figure 2G**). Thus, our results suggest that the *LAMA1* promoter has functional p53-responsive elements and that *LAMA1* expression could directly be transactivated by p53. This is in contrast to LMα5 which is expressed by a p53 independent mechanism.

### Ectopically expressed LMα1 or LMα5 attenuates DSS induced inflammation

**Figure 3:**
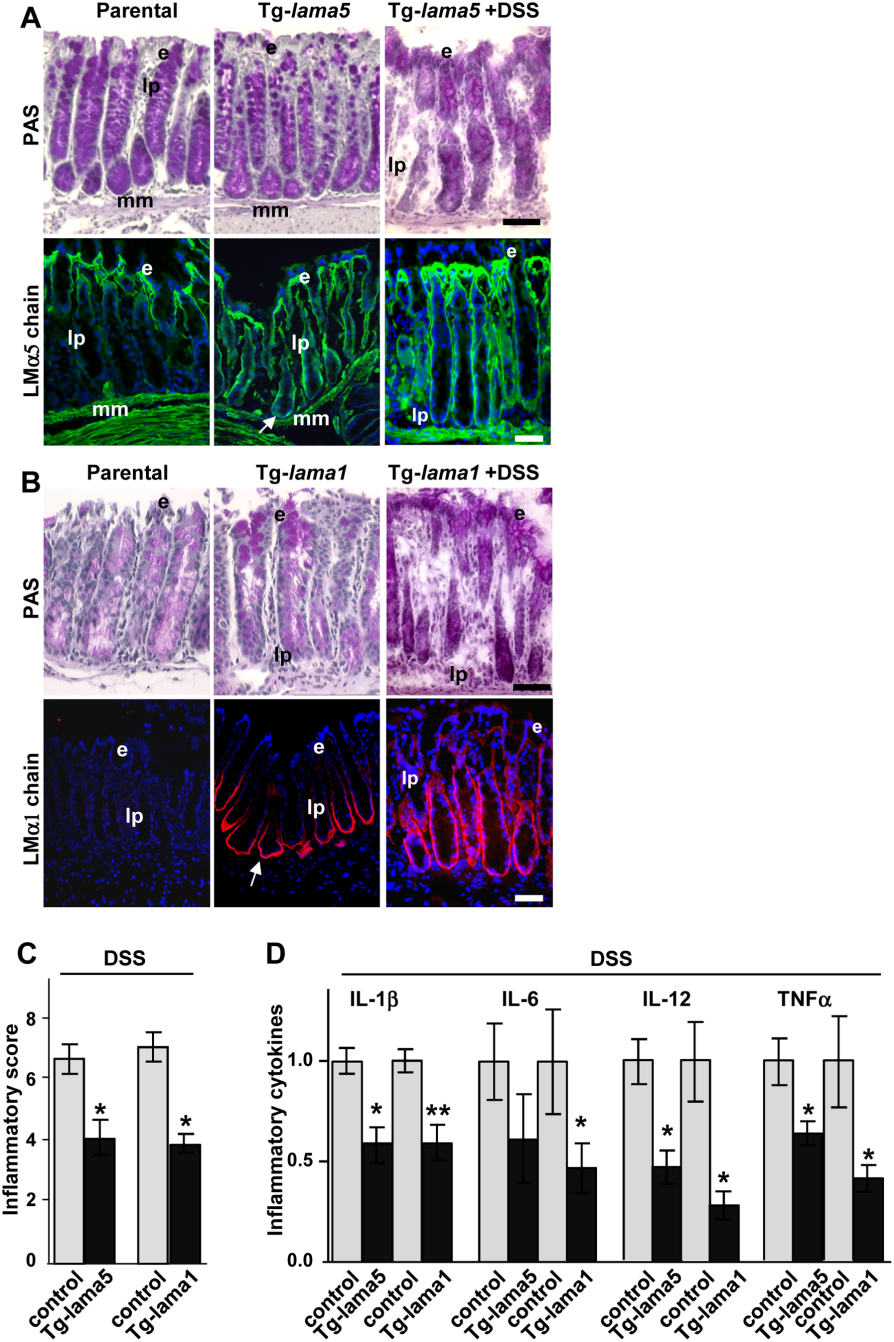
Laminins protect from inflammation. (**A–B**) Histological views of colon tissue (stained with PAS) and expression of LMα5 and LMα1 on cryosections of colon from parental, Tg-*lama5* or Tg-*lama1* mice untreated or treated with DSS. This showed that both chains were ectopically expressed in the glandular crypt region in the transgenic animals (arrows) and that LMα1 expression was further extended by DSS treatment. (**C**) Inflammatory scores (mean +/− SEM; n = 5) assessed on the Swiss-roll of the colon and rectum of transgenic *lama5* and *lama1* animals (black columns) as compared to controls (grey columns), all treated with DSS. (**D**) Levels of pro-inflammatory cytokines (mean +/− SEM; n = 6) measured by ELISA in protein extracts from distal colon of DSS-treated parental (grey columns) and DSS-Tg-*lama1* or DSS-Tg-*lama5* (black columns) mice. Data were normalized to the mean of parental values as levels of cytokines turned out to be mouse strain-dependent. Statistical differences were compared to parental mice. e: epithelial cells; lp: *lamina propria*; mm: *muscularis mucosae*. Nuclei are visualized with DAPI. *p<0.05, **p<0.01. Scale bars: 50 µm.

So far we have described a specific upregulation of LMα1 and LMα5 in IBD and in DSS-induced murine colitis. To assess whether these LM regulate inflammation responses in the intestinal epithelium, transgenic mice that overexpress either LMα1 or LMα5 in the gut (under the intestine-specific villin promoter) were generated ([38], Mammadova-Bach et al., submitted). Immunostaining revealed an ectopic expression of LMα5 (**Figure 3A**) and of LMα1 (**Figure 3B**) at the bottom of the colonic crypts in the respective Tg-*lama5* and Tg-*lama1* animals. The overall structure of the colon was not affected by overexpression of either one of the LM chains (**Figure 3A and 3B**).

To provoke intestinal inflammation adult transgenic mice were subjected to DSS. No further increase in LMα5 staining was noted in colonic mucosa from DSS treated Tg-*lama5* mice as compared to the non-treated animals presumably due to its already high level of expression in the non-inflamed situation (**Figure 3A**). This was different to Tg-*lama1* mice where DSS further increased expression of LMα1 in the BM in two-thirds of the colonic crypts as compared to one half in the DSS-treated wild-type animals (**Figure 3B** **versus** **Figure 1B**). All DSS-treated animals presented signs of distal colitis with variable degree independently of their genotype. Regions of strong inflammation corresponded to significant ulcerations with detachment of the epithelium, presence of distorted glands, abundant stroma and significant infiltration of immune cells. Regions of mild inflammation were defined by an almost normal architecture of the colonic glands in which the epithelium was partially preserved or was in a regenerative state (**Figure S5**). We used staining for tenascin-C (TNC) as marker of inflammation [15], and observed higher TNC expression in the ulcerative tissue (**Figure S5**). Such staining well corresponded to the pattern of TNC expression in human CD or UC colon as published [39], with increased expression at the mucosal surface and in the lamina propria of IBD tissue, concomitantly with increased fibronectin and α-smooth-muscle actin staining (**Figure S7**).

To evaluate whether susceptibility to DSS-induced colitis was potentially affected by the LMα1 and LMα5 expression levels, histological grading of colitis was performed on the Swiss-roll comprising the entire colon and rectum. A detailed assessment of the inflammatory degree was performed. We noticed that the mean score was significantly lower in transgenic mice (4.2 for Tg-*lama5;* 4.0 for Tg-*lama1*) than in controls (6.8 for wt-*lama5* mice; 7.2 for wt-*lama1*) (**Figure 3C**). Therefore, the concentrations of pro-inflammatory cytokines were measured by ELISA in colonic mucosal samples from inflamed parental and transgenic mice. Levels of IL-1β, IL-6, IL-12 and TNFα, classical players implicated in the inflammatory response of DSS-driven colitis, were significantly decreased (up to 3.5-fold) in both Tg-*lama5* and Tg-*lama1* colonic tissue in comparison to controls (**Figure 3D**). NF-κB is a key player known to be implicated in inflammation processes [40]. To test whether LM potentially attenuate NF-κB signaling, HT-29 cells stably expressing a NF-κB reporter [29] were plated on different ECM substrata and were stimulated with TNFα. These experiments revealed that LM-511 was indeed able to attenuate the TNFα-stimulated expression of the NF-κB reporter (**Figure S8A**). As BM are part of the intestinal barrier, we wondered whether an overexpression of a LM chain may physically reinforce the BM. To test the hypothesis that increased levels of LM enhance stiffness of the BM, we performed AFM measurement on matrices deposited by colonic epithelial cells that lacked or expressed the LMα1 chain. Indeed we found that a cell-derived matrix containing the LMα1 chain showed a higher stiffness suggesting a physical BM strenghtening (**Figure S8B**).

Altogether, our data showed that colon inflammation was attenuated in transgenic LMα1 or LMα5 mice as assessed by the histological scoring of inflammation and by decreased expression of inflammatory cytokines, involving regulation of the NF-κB signaling pathway and BM stiffening.

### Impact of LMα1 and LMα5 overexpression on murine colitis-associated tumorigenesis

**Figure 4:**
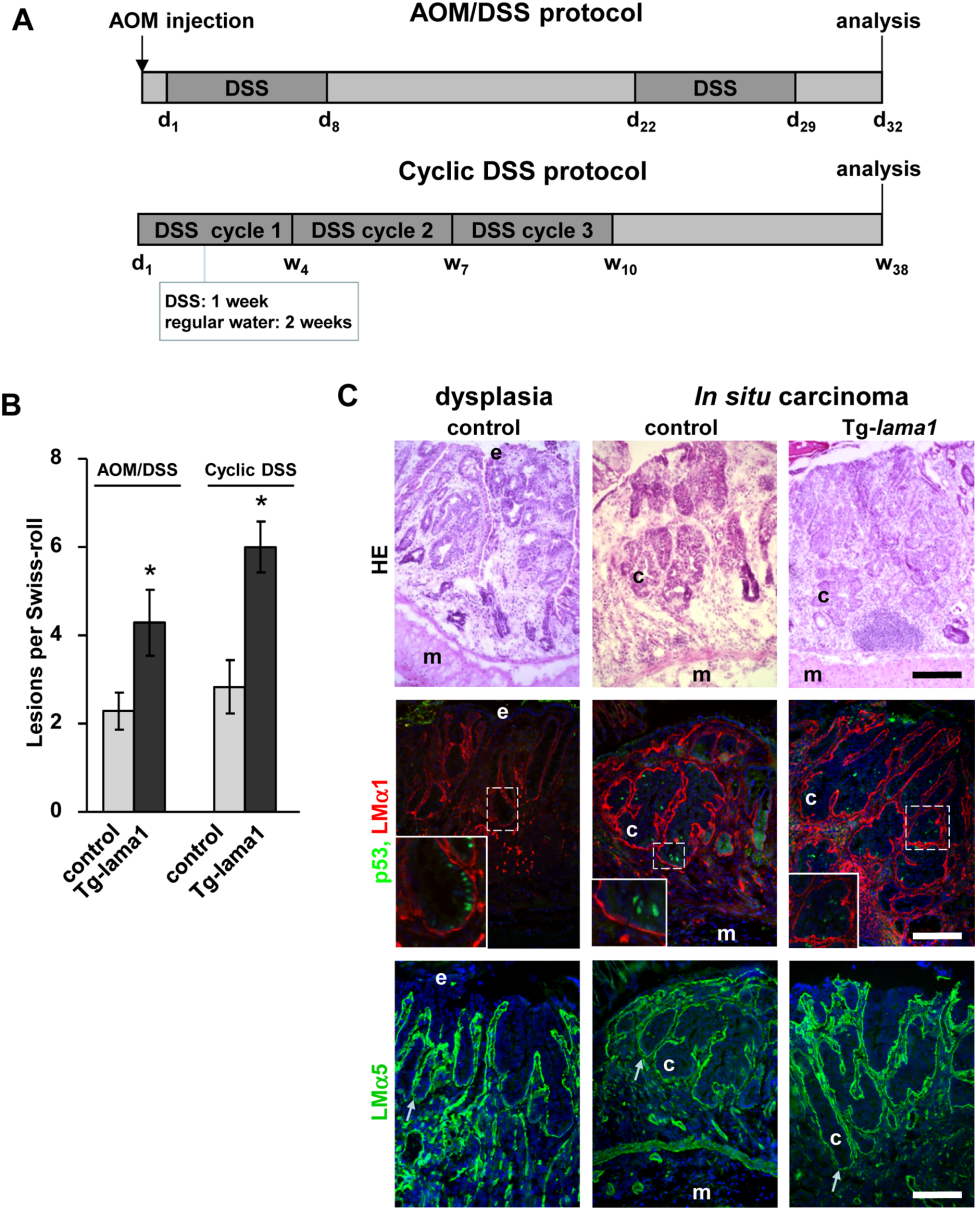
Colitis-associated tumor development is increased in transgenic mice overexpressing LMα1. (**A**) Schematic overview of the AOM/DSS and of the cyclic DSS protocols. (**B**) Control and transgenic mice treated with AOM/DSS or with cyclic DSS develop different types of lesions among them dysplasia and *in situ* carcinomas that were quantified. Tg-*lama1* animals develop more tumors than controls when submitted to the treatments (n = 7, *p<0.05 for AOM/DSS; n = 7, *p<0.05 for cyclic DSS). (**C**) Dysplasia and an *in situ* carcinoma are shown by hematoxylin-eosin staining (HE). Glands in these lesions are strongly positive for LMα1 (red), LMα5 (green, lower panels) and present nuclear p53 (green, middle panels). Nuclei were visualized with DAPI. e: epithelial cells; m: muscle; c: cancer cells; s: stroma; arrows: BM area. Scale bars: 50 µm.

Patients with IBD are at increased risk of developing colitis-associated cancer [8], [41] upon acquisition of oncogenic mutations [42]. Based on our results that had shown that ectopic expression of LMα1 promoted colon tumorigenesis ([31]; Mammadova-Bach et al. submitted) and that high LMα1 and LMα5 expression attenuates colitis associated inflammation (this study) we wondered what impact these LM have on progression of colitis into cancer. Therefore, we first exposed control and transgenic mice to a combined AOM/DSS treatment that, with a short latency period, led to the development of inflammation-driven colorectal tumors due to AOM carcinogen induced mutations [43]. Analysis was performed 3 days after the last DSS treatment (see **Figure 4A**) revealing appearance of dysplastic precursor lesions. After histological examination of the Swiss-rolls we found dysplasia and *in situ* carcinomas that occurred at about a 2-fold higher rate in the colon/rectum region of Tg-*lama1* mice than in controls (**Figure 4B**). The same tendency was also observed in Tg-*lama5* mice although the increase was not statistically significant (p = 0.1336; n = 4; **Figure S9A**). To mimic chronic inflammation that occurs in IBD, we then performed a second colitis-associated cancer model based on cyclic administration of DSS (**Figure 4A**). Comparable to the AOM/DSS model, LMα1 transgenic mice presented also an about 2-fold higher incidence of tumors. Whatever the protocol used, all lesions (dysplasia and *in situ* carcinomas) were characterized by high expression of LMα1 and LMα5 in the BM and by nuclear accumulation of p53 in the epithelial cells (**Figure 4C** and **Figure S9B**).

## Discussion

LM are major components of epithelial BM playing an important role in tissue homeostasis but knowledge regarding their involvement in gastrointestinal pathologies including IBD and colitis-associated cancer remains very limited [26]. The data presented here show that LM can impact on the microenvironmental response to inflammation in the intestine and likely participate in the regeneration process. This is emphasized by an increased LMα1 and LMα5 expression in colon tissues from IBD patients and from DSS-driven colitis in mice. Inflammation was accompanied by a nuclear accumulation of p53 and changes in cell identity/properties as manifested particularly by the presence of UACL in IBD. We provided a mechanistic link between p53 and LM by demonstrating that p53 transactivates *LAMA1* expression through promoter binding. We further showed an attenuated response to DSS-induced inflammation in transgenic mice overexpressing either the LMα1 or LMα5 chain. Yet, overexpression of the same LM molecules could participate in the progression of IBD into colitis-associated cancer upon acquisition of oncogenic mutations as exemplified by AOM/DSS or chronic DSS treated transgenic mice. Our data point to the distinct, sometimes opposing properties of LM, reinforcing their described potential dual functions [44].

Here we showed that in upon inflammation, both LMα1 and LMα5 chains are overexpressed using human IBD and murine colitis specimens. Furthermore we demonstrated in transgenic mice that both LM attenuate DSS-induced inflammation as shown by a reduced inflammatory score and a decreased expression of pro-inflammatory cytokines. These data suggest that α1/α5 chain-containing LM potentially play a role in the IBD disease by limiting colitis. At present time, it was not possible to determine the precise expressed LM isoform, as nobody has managed so far to isolate such thin *in vivo* BM. Yet, the functionality of the LM isoform is known to be mainly mediated by the LMα chain though interaction with cell membrane receptors [45]. Here we provided arguments showing that LMα1 and LMα5 act probably via two distinct (p53 dependent and independent) mechanisms. We first examined a potential involvement of NF-κB because of its documented role in intestinal inflammation [46]. We provided evidence that LM-511 (α5-containing LM) is indeed able to attenuate the TNFα-stimulated expression of the NF-κB reporter. Since LM are constituents of BM which serve as physical and chemical barriers in epithelial tissues it is also possible that their increased abundance in IBD strengthens the BM barrier. Indeed, a cell-derived matrix that contains the LMα1 chain showed an increased stiffness *in vitro*. Altered mechanical properties of LMα1 rich-BM may contribute to protection from inflammation. This hypothesis could be verified in the future owing to the recently developed technology of AFM on isolated BM [47]. Reinforcing BM stability/organization could be a promising therapeutic approach in the early phases of IBD. This might be feasible as a LM substitution “therapy” was already applied to the LMα2 chain-deficient mice where transgenic expression or systemic administration of LM-111 reduced muscular dystrophy [48]. Linked to IBD, reintroduction of colon organoids (embedded in the LM-containing Matrigel) into superficially damaged mouse colon is now feasible [49].

LM could also play a role in tissue restitution as there is some evidence from *in vitro* studies that they promote “wound” closure of disrupted epithelial cell monolayers [50] which is important in tissue rebuilding. This process may be further enhanced by growth factors such as TGFβ and TNFα which have been shown to stimulate LM expression and secretion [25], [51]. In most IBD colon samples we found LMα1 and LMα5 to be highly expressed around UACL that are morphologically and functionally different from the normal colonic crypts. UACL are characterized by defined expression patterns of TFF and mucin molecules [6], [7] and we indeed observed this unusual molecular composition of UACL ([6] and present data) supporting the notion that they participate to repair processes as strengthened previously in the literature [52]. We also found modifications in the expression of transcription factors that play a role in cell fate decision such as Sox9, Pdx1 and Cdx2 which is in accordance to the changes in the pattern of cellular differentiation documented in human IBD [53]. To date, the physiological relevance of this observation remains unclear.

We wondered why and how IBD glands are overexpressing LMα1 and LMα5 and we found that interestingly they also expressed nuclear p53. During the ulceration process, cellular stress and DNA damage occur that typically trigger a p53 response in order to guarantee genome integrity. It is known that active p53 induces a transient cell cycle arrest (absence of Ki67 positive and apoptotic cells in UACL, **Figure S4**) enabling the cell to activate enzymatic DNA repair systems [54]. In this context, we investigated expression of genes implicated in p53 linked DNA repair such as 53BP1, Mlh1, Msh2 and γH2AX (**Table S3**). The first three proteins were expressed in UACL and neighboring glands reflecting a normal response to inflammation and confirming a functional role of nuclear p53 in IBD, while γH2AX was not increased indicative of the absence of DNA double strand lesions.

Besides its role in cell cycle regulation and DNA repair, we suggest a novel function of p53 during IBD by modifying BM properties. Our results suggest that p53 triggers LMα1 expression by binding to the promoter (as evidenced by ChIP assays). This finding does not exclude the possibility that p53 potentially cooperates with other transcriptional regulators such as SP1 that by itself has been shown to induce the murine *lama1* gene [55]. One can postulate that LMα1 could have an indirect positive impact on BM formation by triggering expression of other BM molecules at least *in vivo*. Indeed our present data showed that LMα5 upregulation was independent of p53 and we previously demonstrated that exogenous expression of LMα1 in grafted intestinal HT29 cells had caused increased expression of LMα5 [31]. The concomitant increased of integrin α6β4 ([31] and present study) would argue for a fortified interaction of colonic epithelial cells with their BM. Yet, although LM-111 and LM-511 have been shown to form independent networks under physiological conditions [45], [56], their possible connections and timing of assembly into the BM in IBD and associated-cancer will need to be addressed in the future.

Upon dysregulated ulceration/repair cycles and acquisition of oncogenic alterations, IBD could degenerate into cancer [57]. To mimic IBD-associated cancer we developed two models of colitis-associated tumorigenesis in transgenic LM-overexpressing mice. We showed that chronic DSS- and AOM/DSS-induced tumor formation was enhanced in the intestine of LMα1 transgenic mice suggesting that in a carcinogenic context LMα1 favors tumorigenesis. Mutations of p53 have been described as an early event in colitis-associated cancer [42] and more recently it was also demonstrated that mutated p53 promotes progression of IBD into associated colon cancer [13]. In the murine model we found nuclear p53 both in dysplasia and in tumors. The status of p53, whether mutated or not, is unknown in our samples. Whether and how ectopic LMα1 and LMα5 are organized into BM in IBD and in particular during colitis associated cancer is important to elucidate and might provide novel means to fight cancer.

**Figure 5:**
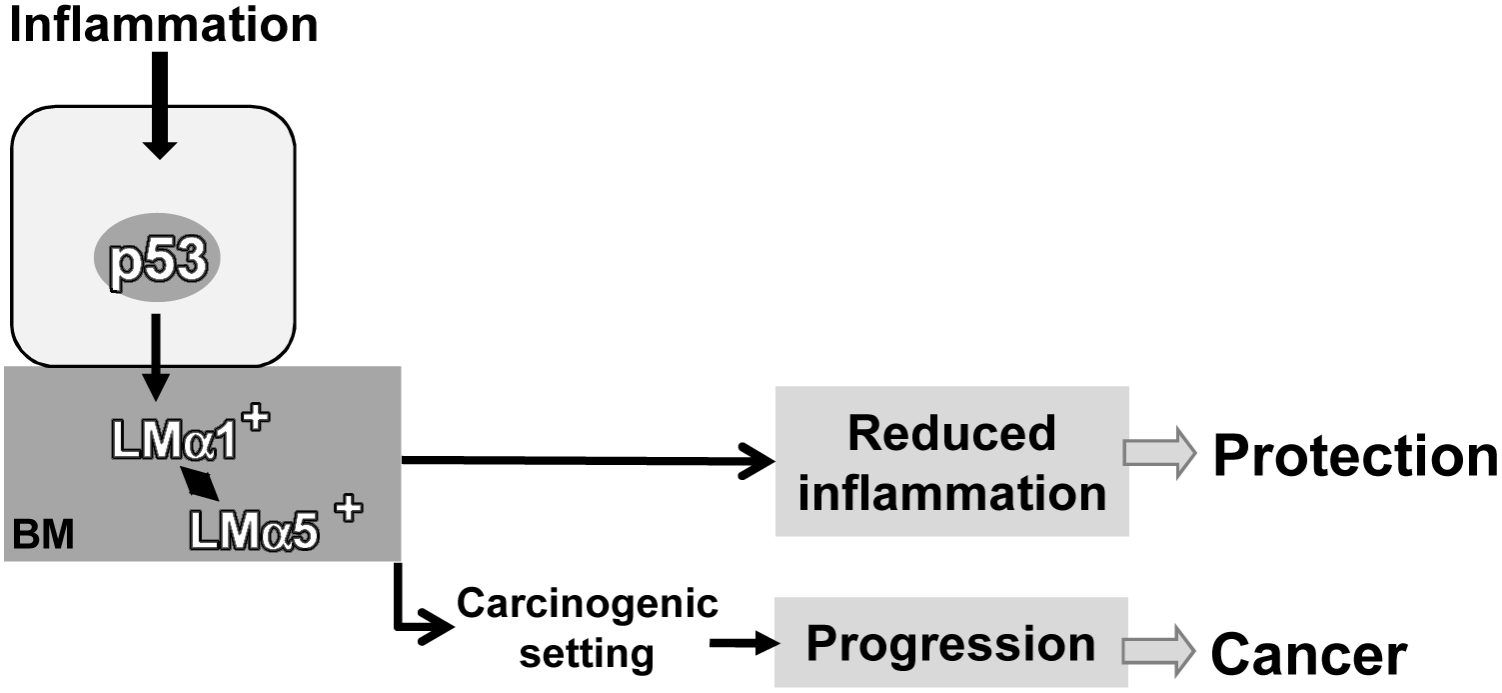
Important role of p53 in regulating LM expression in IBD and colitis-associated cancer. We propose the following scenario where inflammation triggers nuclear accumulation of p53 that transactivates expression of LMα1 and its deposition to the BM concomitantly to that of LMα5. Overexpression of LMα5 in response to inflammation is a p53 independent mechanism. In a chronically inflamed microenvironment highly expressed LMα1 and LMα5 may provide physical barrier function resulting in attenuated inflammation as demonstrated in transgenic mice. Yet, high LMα1 and LMα5 in a carcinogenic setting subsequent to chronic colitis may contribute to a pro-tumorigenic microenvironment.

Taken together our results showed that the forced expression of LMα1 and LMα5 (which are elevated in human colitis) protected against DSS-induced inflammation. But in carcinogenic conditions the same LM molecules accelerate colitis-associated tumorigenesis (**Figure 5**). More knowledge about the switch from good (reparation) into evil (cancer) is required where our transgenic mice represent attractive new models. In the early phases of IBD, reinforcing BM stability may be a promising therapeutic approach.

## Supporting Information

Figure S1 **Assessment of the inflammatory scores in Crohn’s disease and ulcerative colitis.** (**A**) The macroscopic appearances of the colonic mucosa after hematoxylin-eosin staining were graded using the Riley’s score on 25 Crohn patients and 7 Ulcerative colitis patients giving a mean value ranging from 8.3 to 8.6 while control samples never exceed 1.4. (**B**) Inflammation was further confirmed by immunohistochemistry showing strong CD45 and TLR4 reactivity in samples from CD patients. Scale bars: 25 µm.
(TIF)

Figure S2 **Spatial distribution of LMα2, LMα3 and LMα4 chains in non-inflamed and inflamed colon tissues from IBD patients.** Representative immunofluorescence pictures for LMα2, LMα3 and LMα4 showing the presence of LMα2 in crypt glands (arrowheads) and around UACL for LMα2 and LMα3. Note that anti-LMα4 antibodies stained the myofibroblasts (inset) located underneath the BM as well as the muscularis mucosae. Nuclei are visualized with DAPI. e: epithelial cells; lp: *lamina propria*; mm: *muscularis mucosae*; g: aspecific staining of mucus cells; arrows: BM staining around UACL. Scale bar: 50 µm.
(TIF)

Figure S3 **Characteristic features of the UACL found in IBD patients.** (**A**) The UACL were identified as glandular structures strongly stained in magenta with PAS as compared to the normal colonic glands; they were characterized by elongated flat nuclei at the basal pole of the cell (inset; staining with hematoxylin-eosin). (**B**) The cells that composed the UACL showed immunoreactivity for MU5AC (inset) and MUC6, while no reactivity was observed for MUC2 or Cdx2 as compared to normal colonic areas; UACL are positive or negative for TFF1 and TFF3 depending on their location within the mucosa. Nuclei are visualized with DAPI. Arrows point to the UACL. * points to morphologically-defined atypical glands slightly stained for TFF1. Scale bars: 50 µm.
(TIF)

Figure S4 **Immunohistochemical characterization of the UACL lineage including proliferation, apoptosis, cell identity markers and repair proteins.** The cells that composed the UACL in IBD do not proliferate (negative immunostaining for Ki67) and do not reveal sign of apoptosis (TUNEL assay). They still show positive immunoreactivity for epithelial markers (E-cadherin, cytokeratin-19) and display expression of repair proteins (Mlh1 and Msh2). Nuclei of the UACL are positive for actors of the Wnt pathway such as β-catenin, c-myc and Sox9. The presence of the Pdx1 transcription factor in the nuclei of UACL combined to the mucin and TFF profiles (depicted in **Figure S3**) are features of gastro-duodenal metaplasia. Arrows point to the UACL. Scale bar: 50 µm.
(TIF)

Figure S5 **Analysis of inflammation on Swiss-rolls of the distal intestine.** Representative picture (HE) of a cryosection throughout a Swiss-roll of colon and rectum from a wt mouse treated with DSS; the proximal colon is located on the external part of the Swiss-roll while the rectum is at the centre. (1–3) Enlargement of representative zones found along the Swiss-roll: (1) area corresponding to strong signs of ulceration with distorted/altered glands where there was accumulation of tenascin-C (TNC) in the stroma and in the apical region of the glands; (2) region of mild inflammation where the colonic mucosa was partially preserved showing also an increased TNC staining in the stroma; (3) non-inflamed mucosa showing the typical TNC staining at the upper part of the gland.
(TIF)

Figure S6 **Expression of integrin α6 and β4 subunits in human and mouse presenting colitis.** (**A**) In human, IBD colon samples presented an increased staining of α6 and β4 integrins at the bottom of the gland and a strong immunoreactivity mostly confined to the basal part of epithelial cells from the UACL in contrast to normal adjacent glands (a) where lateral staining was also observed. (**B**) Such increased staining of α6 and β4 integrins was also obvious in murine colitis tissue. Nuclei are visualized with DAPI. e: epithelial cells; lp: *lamina propria.* Scale bars: 50 µm.
(TIF)

Figure S7 **Analysis of the stroma and myofibroblasts in colon from IBD patients.** Representative micrographs from colonic control, IBD specimens and from UACL obtained after immunofluorescence staining for TNC, fibronectin (FN) or by immunohistochemistry for the detection of α-smooth muscle actin (α-SM actin, marker of activated fibroblasts). TNC staining was increased at the mucosal surface and in the lamina propria (asterisk) of IBD samples, especially in UC patients as well as around the UACL. Similarly, FN and α-SM actin were also upregulated in the stromal compartment of IBD samples as well as around UACL. Nuclei are visualized with DAPI. e: epithelial cells; lp: *lamina propria*; mm: *muscularis mucosae*; arrows: UACL Scale bars: 50 µm.
(TIF)

Figure S8 **LM-511 inhibits the inflammatory response to TNFα via NF-κB and LM-111 reinforces the BM by increasing its stiffness.** (**A**) NF-κB reporting HT-29 cells were cultured with or without TNFα on different matrix substrata and compared to dishes without matrix (no coating). The values are given as fold change in luciferase activity (ratio with TNFα/without TNFα; mean +/− SEM from 6 independent experiments). Note that LM-511 inhibits the TNFα-stimulated expression of the reporter gene assessed by luciferase activity as compared to other ECM molecules. (**B**) Young’s modulus (E, in Pa) was calculated after AFM measurements of the cell-derived matrix expressing (+LMα1) or not (control) the LMα1 chain reflecting its stiffness. The values are given as mean +/− SEM from 25 measurements of 3 different areas of 50×50 µm in each dish. Note that the stiffness of the cell-derived matrix expressing LMα1 was statistically increased as compared to the LMα1-deprived matrix. Coll. I: collagen I; ***p<0.001.
(TIF)

Figure S9 **Colitis-associated tumor development in transgenic overexpressing LM mice.** (**A**) Dysplasia and *in situ* carcinoma were determined in Swiss-roll from Tg-*lama5* and control mice upon treatment with AOM/DSS. Similarly to *Tg-lama1* mice more tumors have been found in Tg-*lama5* colon than control animals although not statistically significant (mean +/− SEM; n = 4; p = 0.1336). Note that Tg-*lama5* and control littermates develop fewer lesions than Tg-*lama1* (see **Figure 4**) probably due to genetic background differences. (**B**) Likewise to the AOM/DSS treatment, dysplasia and *in situ* carcinoma have been induced by cyclic DSS treatment in *Tg-lama1* mice. The lesions presented high LMα1 and LMα5 expression at the interface between cancer cells and stroma. p53 staining was obvious in numerous nuclei. e: epithelial cells; c: cancer cells; arrows: BM area.
(TIF)

Table S1 **Description of the antibodies used.**
(PDF)

Table S2 **Sequences of the primers used for genotyping, RT-qPCR and chromatin immunoprecipitation.**
(PDF)

Table S3 **List of the markers examined to characterize the UACL.**
(PDF)

Methods S1 **Mouse models; Histology and immunodetection; Cell culture conditions; quantitative RT-PCR.**
(PDF)
