# Supplementary figures and images for: The Laminin Response in Inflammatory Bowel Disease: Protection or Malignancy?
Source: PLoS One. 2014 Oct 27;9(10):e111336. doi: 10.1371/journal.pone.0111336 (PMC4210184; doi:10.1371/journal.pone.0111336)

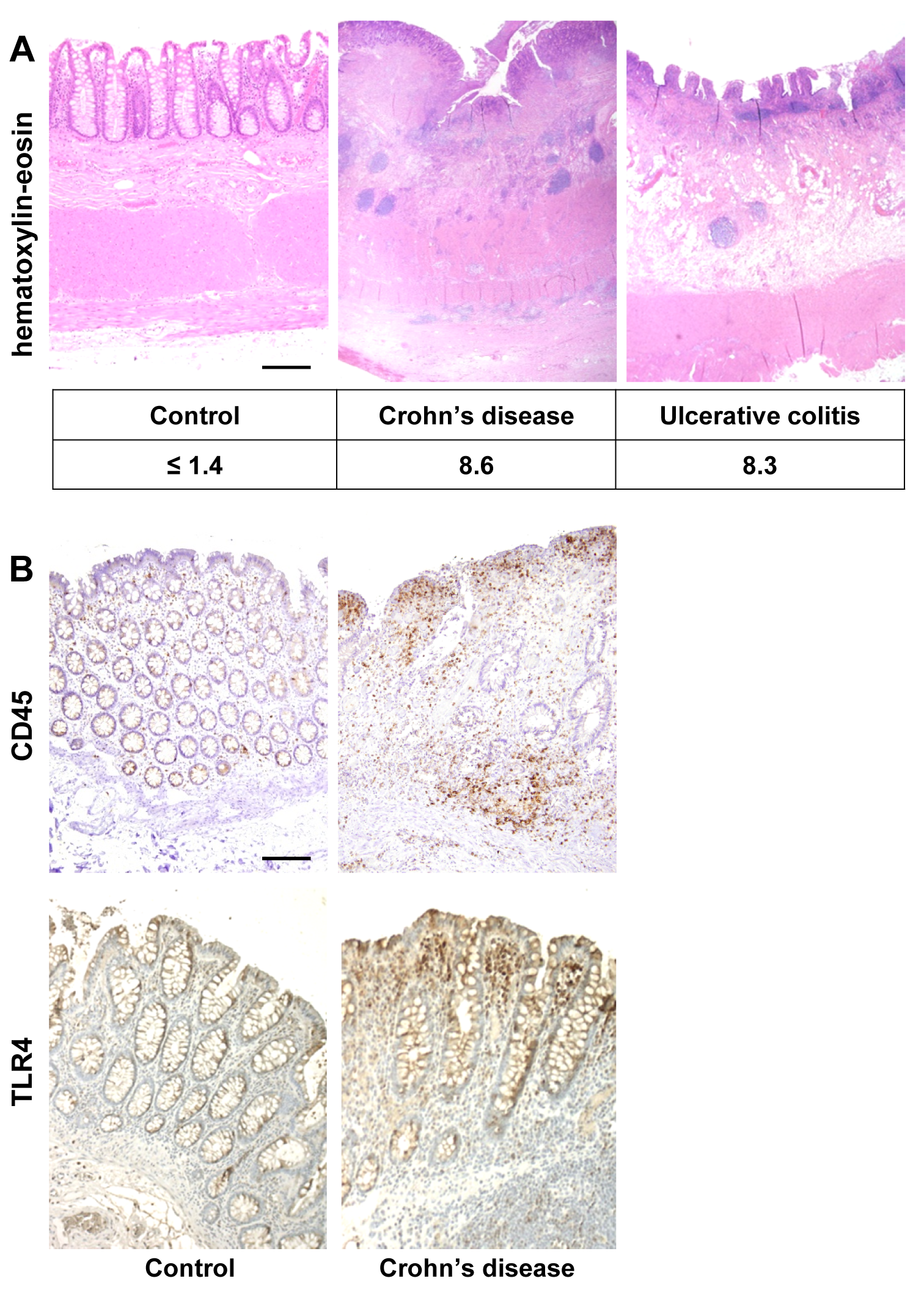

Supplement: Figure S1 — Assessment of the inflammatory scores in Crohn’s disease and ulcerative colitis. (A) The macroscopic appearances of the colonic mucosa after hematoxylin-eosin staining were graded using the Riley’s score on 25 Crohn patients and 7 Ulcerative colitis patients giving a mean value ranging from 8.3 to 8.6 while control samples never exceed 1.4. (B) Inflammation was further confirmed by immunohistochemistry showing strong CD45 and TLR4 reactivity in samples from CD patients. Scale bars: 25 µm. (TIF) [file pone.0111336.s001.tif]

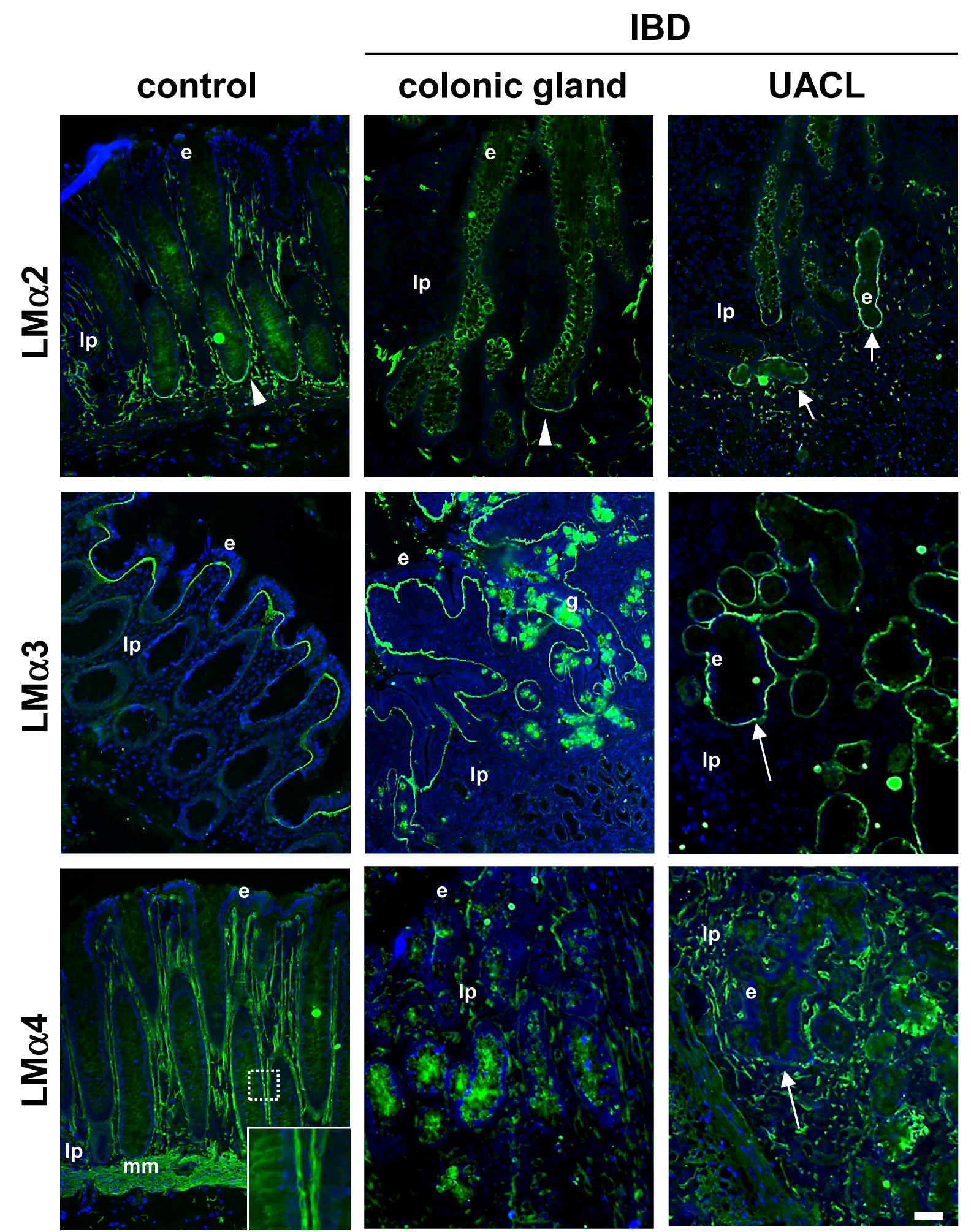

Supplement: Figure S2 — Spatial distribution of LMα2, LMα3 and LMα4 chains in non-inflamed and inflamed colon tissues from IBD patients. Representative immunofluorescence pictures for LMα2, LMα3 and LMα4 showing the presence of LMα2 in crypt glands (arrowheads) and around UACL for LMα2 and LMα3. Note that anti-LMα4 antibodies stained the myofibroblasts (inset) located underneath the BM as well as the muscularis mucosae. Nuclei are visualized with DAPI. e: epithelial cells; lp: lamina propria; mm: muscularis mucosae; g: aspecific staining of mucus cells; arrows: BM staining around UACL. Scale bar: 50 µm. (TIF) [file pone.0111336.s002.tif]

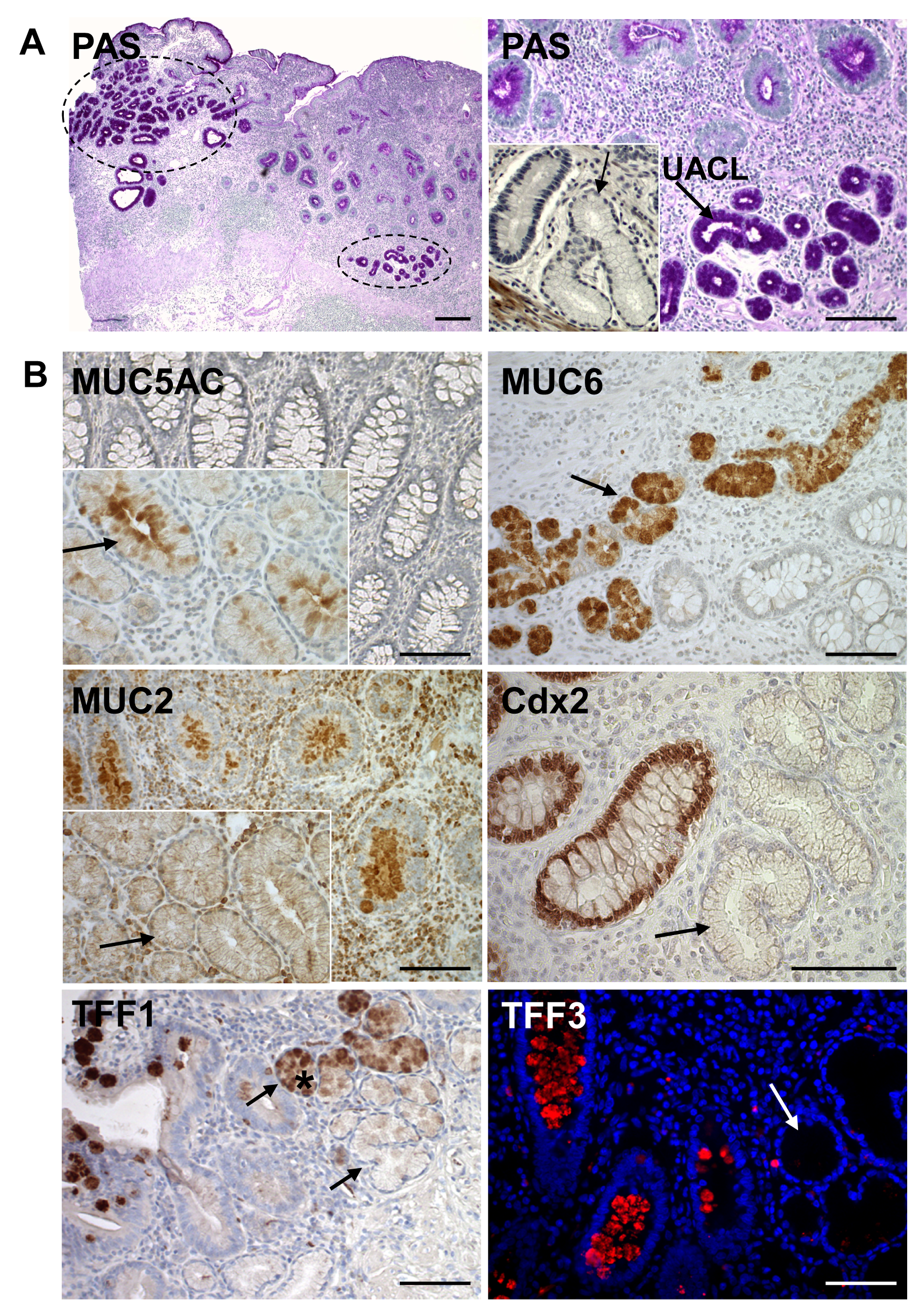

Supplement: Figure S3 — Characteristic features of the UACL found in IBD patients. (A) The UACL were identified as glandular structures strongly stained in magenta with PAS as compared to the normal colonic glands; they were characterized by elongated flat nuclei at the basal pole of the cell (inset; staining with hematoxylin-eosin). (B) The cells that composed the UACL showed immunoreactivity for MU5AC (inset) and MUC6, while no reactivity was observed for MUC2 or Cdx2 as compared to normal colonic areas; UACL are positive or negative for TFF1 and TFF3 depending on their location within the mucosa. Nuclei are visualized with DAPI. Arrows point to the UACL. * points to morphologically-defined atypical glands slightly stained for TFF1. Scale bars: 50 µm. (TIF) [file pone.0111336.s003.tif]

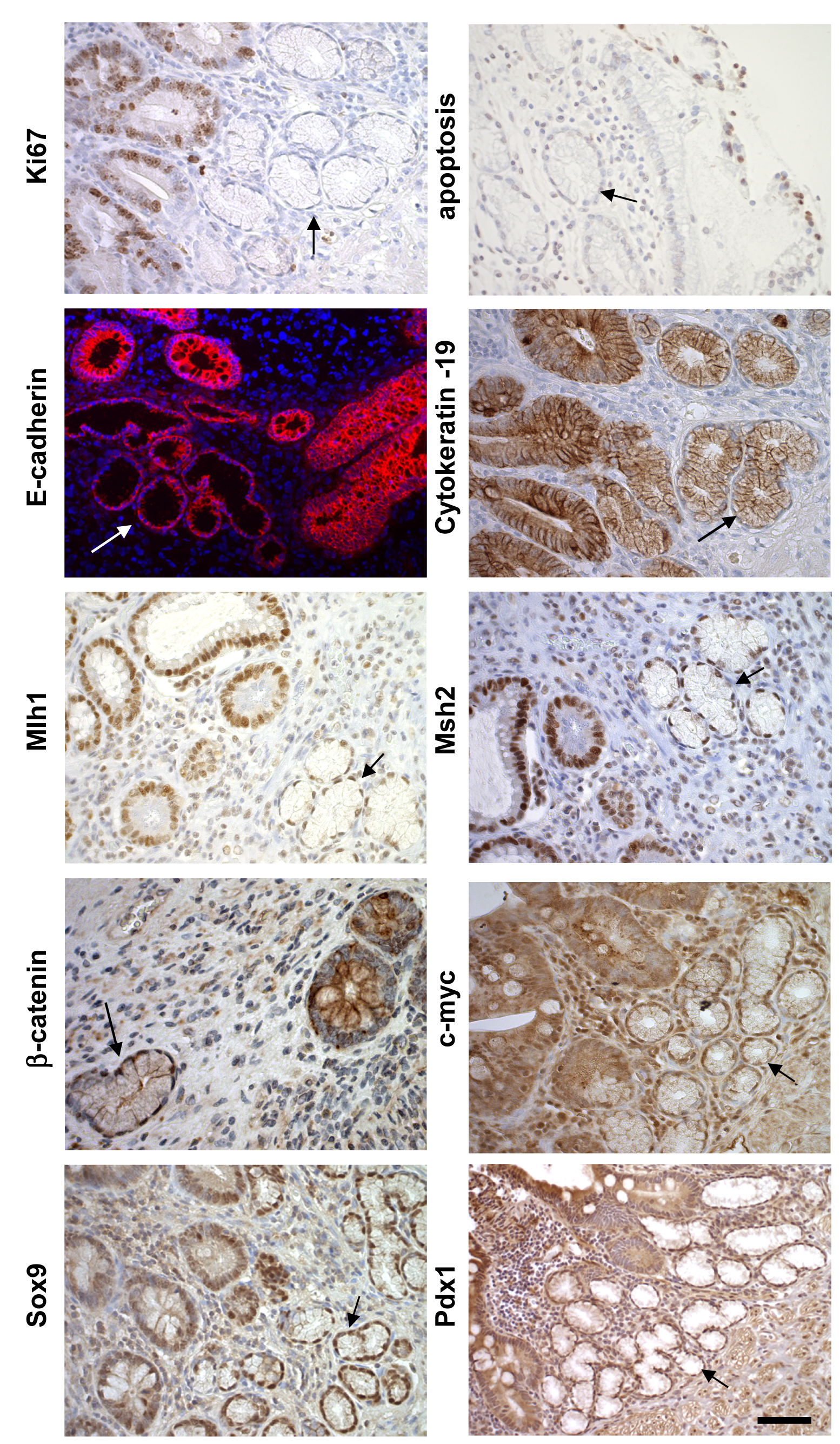

Supplement: Figure S4 — Immunohistochemical characterization of the UACL lineage including proliferation, apoptosis, cell identity markers and repair proteins. The cells that composed the UACL in IBD do not proliferate (negative immunostaining for Ki67) and do not reveal sign of apoptosis (TUNEL assay). They still show positive immunoreactivity for epithelial markers (E-cadherin, cytokeratin-19) and display expression of repair proteins (Mlh1 and Msh2). Nuclei of the UACL are positive for actors of the Wnt pathway such as β-catenin, c-myc and Sox9. The presence of the Pdx1 transcription factor in the nuclei of UACL combined to the mucin and TFF profiles (depicted in Figure S3) are features of gastro-duodenal metaplasia. Arrows point to the UACL. Scale bar: 50 µm. (TIF) [file pone.0111336.s004.tif]

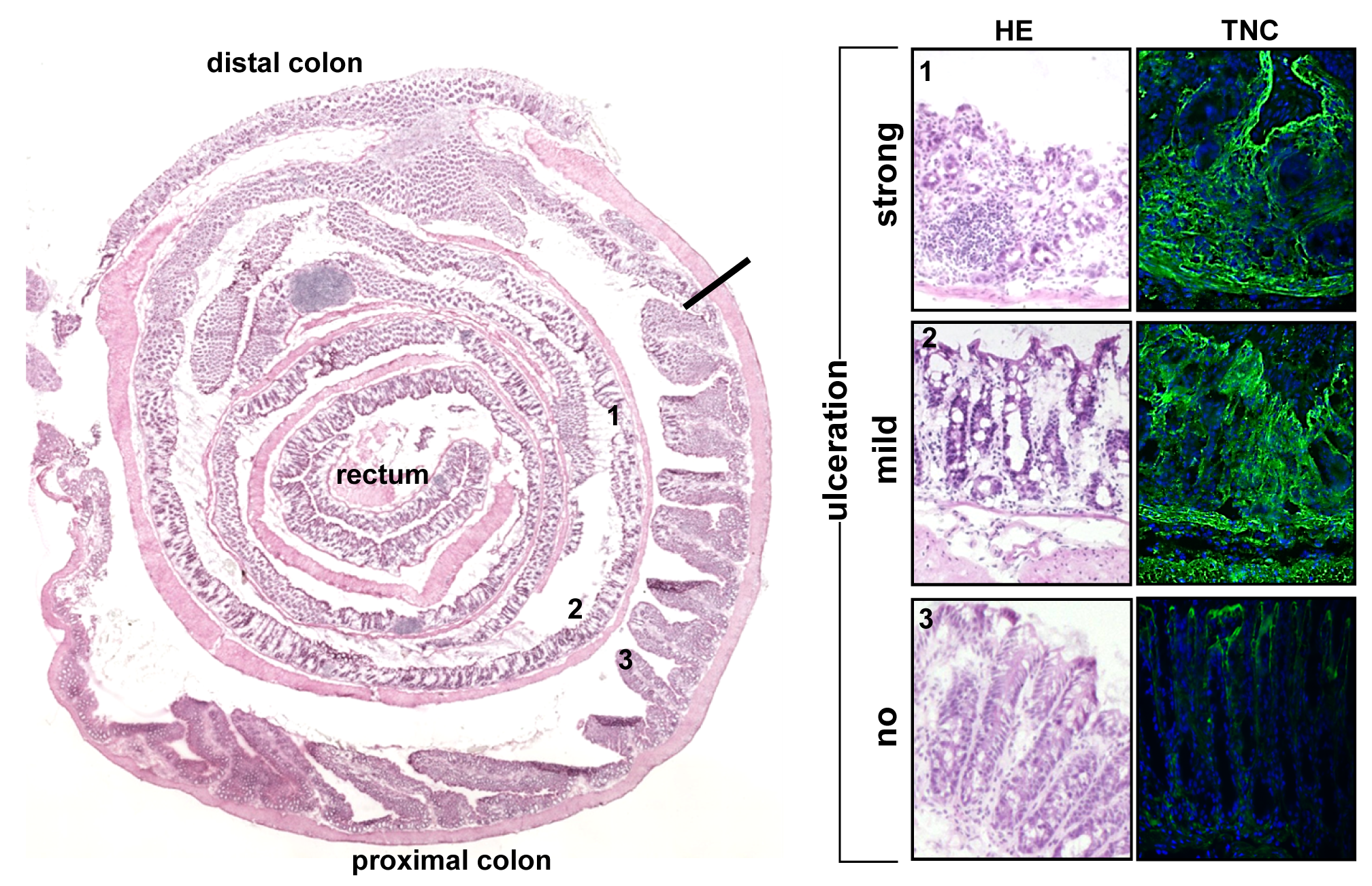

Supplement: Figure S5 — Analysis of inflammation on Swiss-rolls of the distal intestine. Representative picture (HE) of a cryosection throughout a Swiss-roll of colon and rectum from a wt mouse treated with DSS; the proximal colon is located on the external part of the Swiss-roll while the rectum is at the centre. (1–3) Enlargement of representative zones found along the Swiss-roll: (1) area corresponding to strong signs of ulceration with distorted/altered glands where there was accumulation of tenascin-C (TNC) in the stroma and in the apical region of the glands; (2) region of mild inflammation where the colonic mucosa was partially preserved showing also an increased TNC staining in the stroma; (3) non-inflamed mucosa showing the typical TNC staining at the upper part of the gland. (TIF) [file pone.0111336.s005.tif]

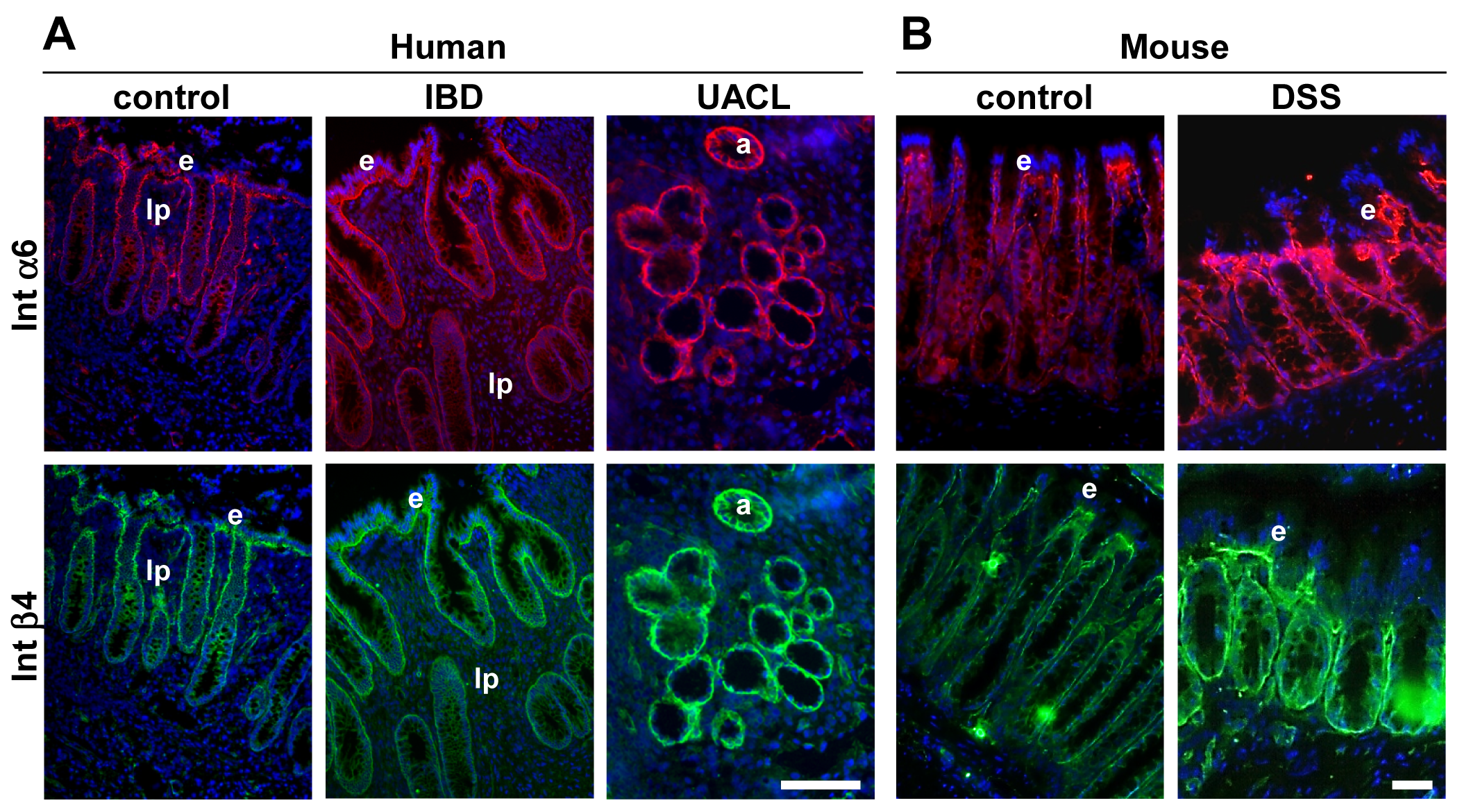

Supplement: Figure S6 — Expression of integrin α6 and β4 subunits in human and mouse presenting colitis. (A) In human, IBD colon samples presented an increased staining of α6 and β4 integrins at the bottom of the gland and a strong immunoreactivity mostly confined to the basal part of epithelial cells from the UACL in contrast to normal adjacent glands (a) where lateral staining was also observed. (B) Such increased staining of α6 and β4 integrins was also obvious in murine colitis tissue. Nuclei are visualized with DAPI. e: epithelial cells; lp: lamina propria. Scale bars: 50 µm. (TIF) [file pone.0111336.s006.tif]

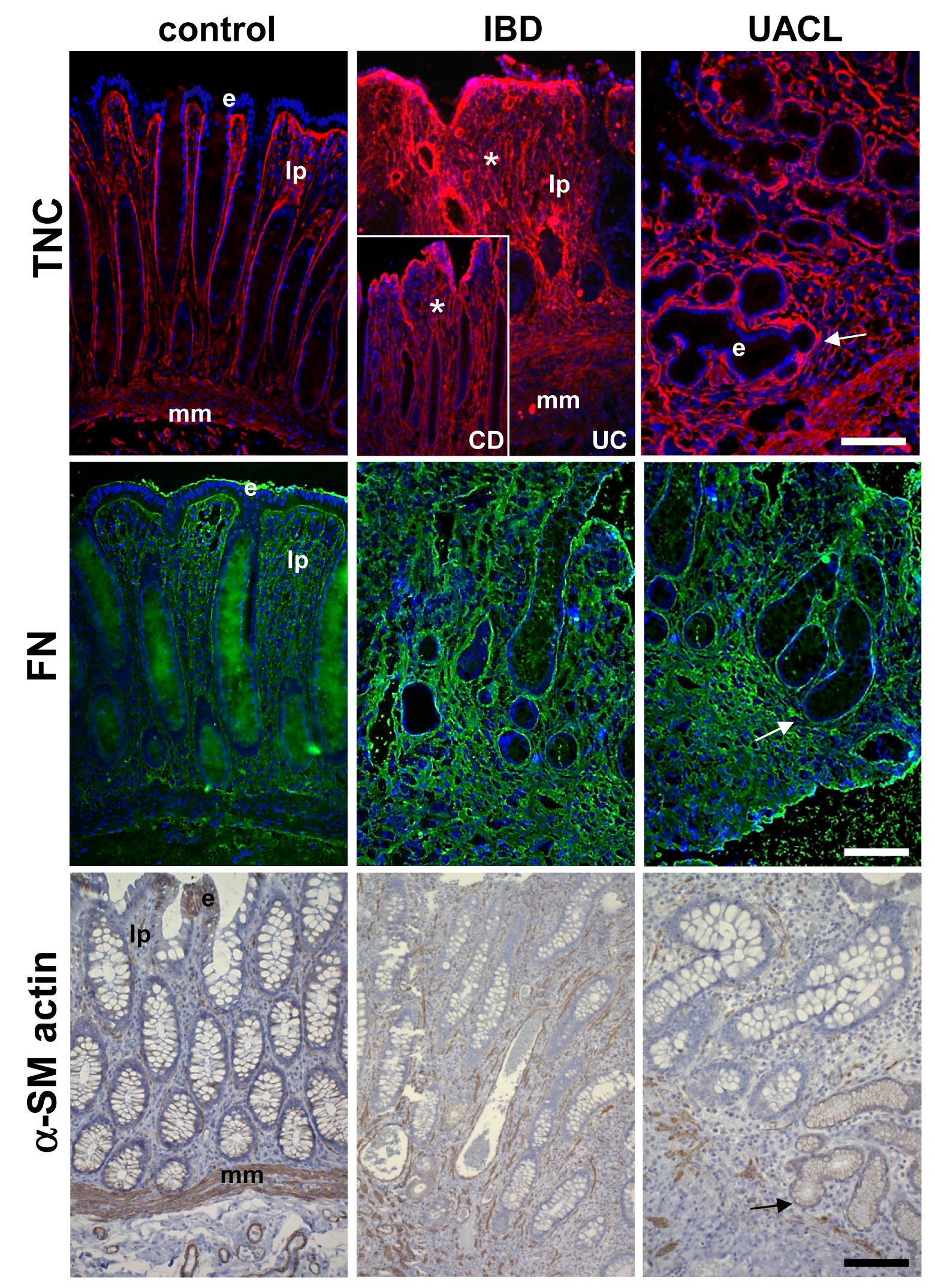

Supplement: Figure S7 — Analysis of the stroma and myofibroblasts in colon from IBD patients. Representative micrographs from colonic control, IBD specimens and from UACL obtained after immunofluorescence staining for TNC, fibronectin (FN) or by immunohistochemistry for the detection of α-smooth muscle actin (α-SM actin, marker of activated fibroblasts). TNC staining was increased at the mucosal surface and in the lamina propria (asterisk) of IBD samples, especially in UC patients as well as around the UACL. Similarly, FN and α-SM actin were also upregulated in the stromal compartment of IBD samples as well as around UACL. Nuclei are visualized with DAPI. e: epithelial cells; lp: lamina propria; mm: muscularis mucosae; arrows: UACL Scale bars: 50 µm. (TIF) [file pone.0111336.s007.tif]

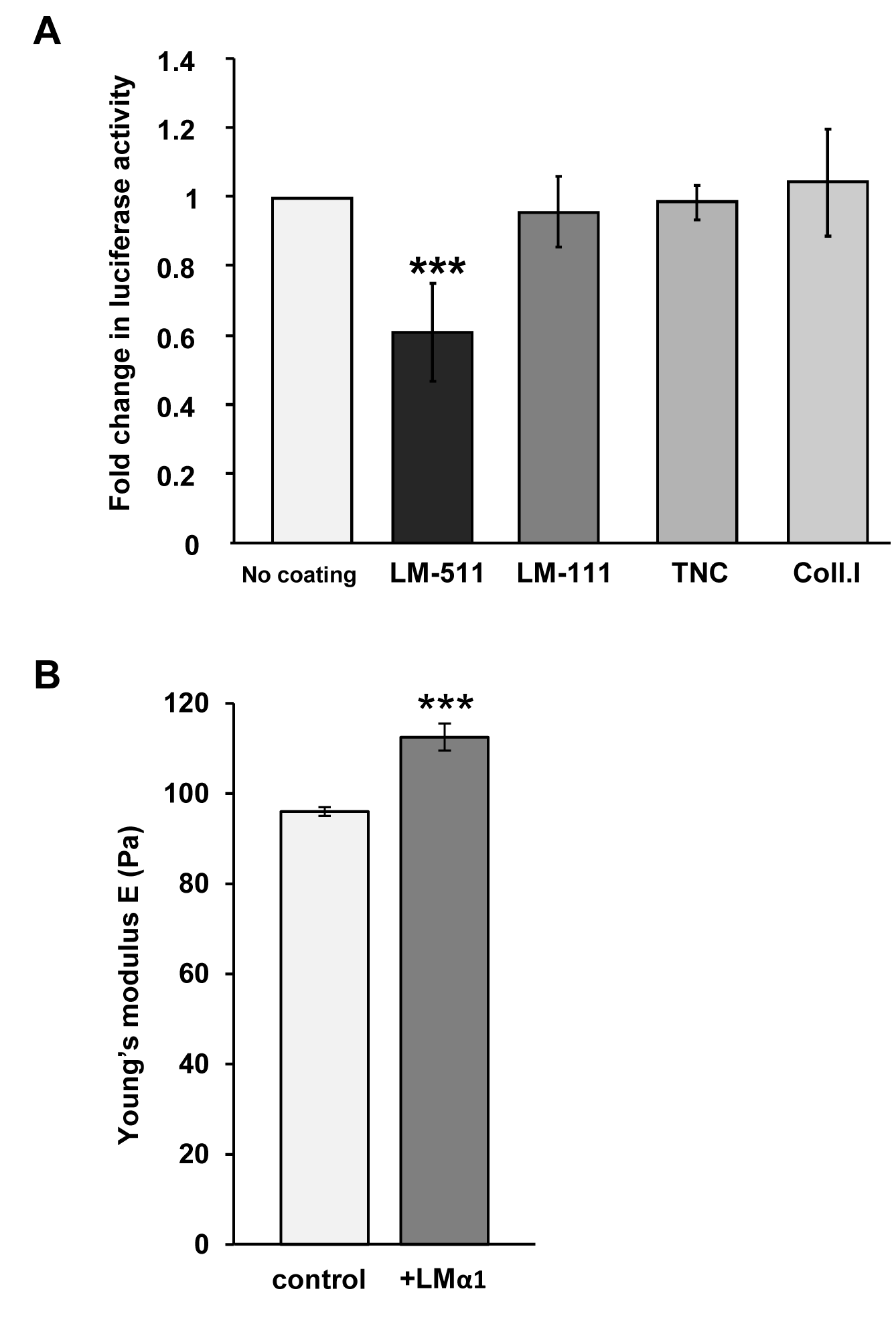

Supplement: Figure S8 — LM-511 inhibits the inflammatory response to TNFα via NF-κB and LM-111 reinforces the BM by increasing its stiffness. (A) NF-κB reporting HT-29 cells were cultured with or without TNFα on different matrix substrata and compared to dishes without matrix (no coating). The values are given as fold change in luciferase activity (ratio with TNFα/without TNFα; mean +/− SEM from 6 independent experiments). Note that LM-511 inhibits the TNFα-stimulated expression of the reporter gene assessed by luciferase activity as compared to other ECM molecules. (B) Young’s modulus (E, in Pa) was calculated after AFM measurements of the cell-derived matrix expressing (+LMα1) or not (control) the LMα1 chain reflecting its stiffness. The values are given as mean +/− SEM from 25 measurements of 3 different areas of 50×50 µm in each dish. Note that the stiffness of the cell-derived matrix expressing LMα1 was statistically increased as compared to the LMα1-deprived matrix. Coll. I: collagen I; ***p<0.001. (TIF) [file pone.0111336.s008.tif]

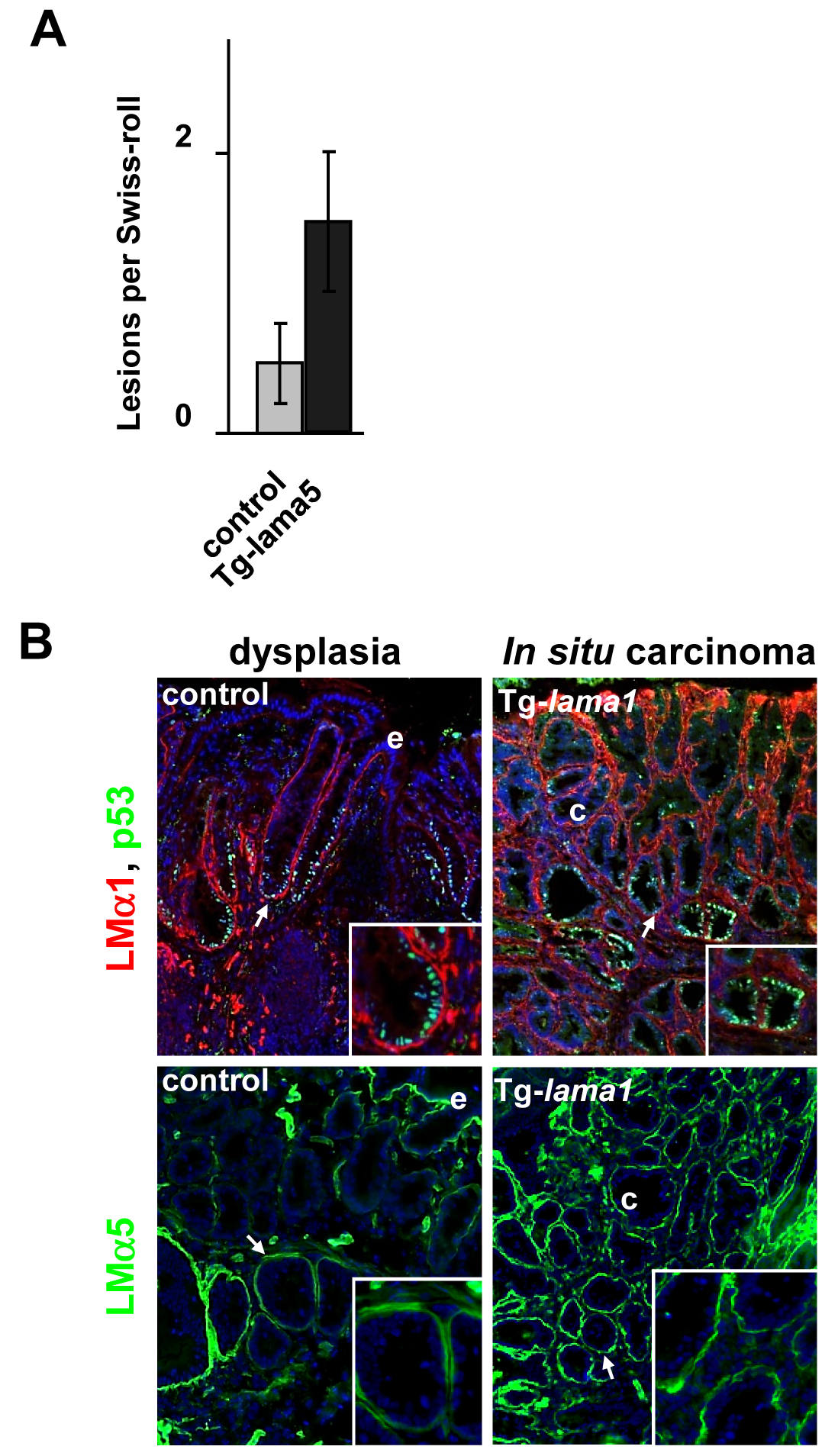

Supplement: Figure S9 — Colitis-associated tumor development in transgenic overexpressing LM mice. (A) Dysplasia and in situ carcinoma were determined in Swiss-roll from Tg-lama5 and control mice upon treatment with AOM/DSS. Similarly to Tg-lama1 mice more tumors have been found in Tg-lama5 colon than control animals although not statistically significant (mean +/− SEM; n = 4; p = 0.1336). Note that Tg-lama5 and control littermates develop fewer lesions than Tg-lama1 (see Figure 4 ) probably due to genetic background differences. (B) Likewise to the AOM/DSS treatment, dysplasia and in situ carcinoma have been induced by cyclic DSS treatment in Tg-lama1 mice. The lesions presented high LMα1 and LMα5 expression at the interface between cancer cells and stroma. p53 staining was obvious in numerous nuclei. e: epithelial cells; c: cancer cells; arrows: BM area. (TIF) [file pone.0111336.s009.tif]
