# Supplementary material for: The Laminin Response in Inflammatory Bowel Disease: Protection or Malignancy?
Source: PLoS One. 2014 Oct 27;9(10):e111336. doi: 10.1371/journal.pone.0111336 (PMC4210184; doi:10.1371/journal.pone.0111336)
Supplement: Table S1 — Description of the antibodies used. (PDF) [file pone.0111336.s010.pdf]

specificities	host	antibody name	source	dilution	purpose
Laminin α1	rabbit anti-human	α1 G4/G5	Dr D Gullberg	1/400	IF
Laminin α1	rat anti-mouse	200	Dr L Sorokin	1/100	IF
Laminin α2	mouse anti-human	2D10/E7	Dr E Engvall	1/50	IF
Laminin α3	mouse anti-human	BM2	Dr P Rousselle	1/100	IF
Laminin α4	mouse anti-human	3D12	Dr L Sorokin	pure	IF
Laminin α5	mouse anti-human	4C7	Dr E Engvall	1/100	IF
Laminin α5	rabbit anti-mouse	405	Dr L Sorokin	1/500	IF
Laminin β1	mouse anti-human	IIID9	Dr L Sorokin	pure	IF
Laminin β2	mouse anti-human	9F8	Dr L Sorokin	pure	IF
Laminin γ1	mouse anti-human	IIID10	Dr L Sorokin	pure	IF
Laminin γ2	rabbit anti-human	2140	Dr P Simon-Assmann	1/1000	IF
Integrin α6	rat	GoH3	Immunotech	1/50	IF
Integrin β4	mouse	1964-3E1	Euromedex	1/1000	IF
Integrin β1	mouse anti-human	P4C10	Gibco BRL	1/1000	IF
TNC	rabbit	TNC 1.2	Dr G Orend	1/200	IF
FN	rabbit	FN 2.1	Dr G Orend	1/200	IF
MUC2	rabbit anti-human	1A + 2B MUC2	Dr J Bara	1/200	IHC
MUC2	rabbit anti-mouse	sc-15334	Santa Cruz Biotechnology	1/1000	IF
MUC5AC	mouse	PM9	Dr J Bara	pure	IHC
MUC6	mouse	F8	Dr J Bara	pure	IHC
TFF1	mouse	P2802	Dr C Tomasetto	1/100	IF
TFF3	rabbit	1519	Dr C Tomasetto	1/500	IF
Cdx2	mouse	CDX2-88	BioGenex	1/500	IHC
p53	mouse anti-human	DO-7	Dako	1/200	IHC V
p53	rabbit anti-mouse	CM5	Novocastra	1/100	IF
p53	mouse	DO-1	Santa Cruz Biotechnology	1µg/10^6 ¢	IP
p53	mouse	PAb 1801	BD Pharmingen	1µg/10^6 ¢<br>1/1000	IP<br>WB
Actin	mouse	C4	Chemicon	1/30000	WB
Mdm2	mouse anti-human	IF2	Invitrogen	1/500	IHC
Bcl-2	mouse	clone 124	Dako	1/100	IHC V
E-cadherin	mouse	clone 36	BD Transduction Laboratories™	1/1000	IF
β-catenin	mouse	clone 14	BD Transduction Laboratories™	1/100	IHC
α-SM1	mouse	clone 1A4	Dako	1/300	IHC V
Msh2	mouse	G219-1129	Ventana	prediluted	IHC V
Mlh1	mouse	G168-728	Ventana	predilited	IHC V
Ki67	mouse	MM1	Novocastra	1/200	IHC
TLR4	rabbit	CD284	Imgenex	1/50	IHC
CD31	mouse	MEM-05	Invitrogen	1/200	IF
CD45	mouse	2B11+PD7/26	Dako	1/100	IHC V
CD68	mouse	KP1	Dako	1/4000	IHC V
Pdx1	rabbit	-	Dr C Wright	1/2000	IHC
Sox9	rabbit	AB5535	Millipore	1/1000	IHC
γH2AX	mouse	JBW301	Millipore	1/100	IHC
53BP1	rabbit	NB100-304	Novus Biologicals	1/100	IHC
EGFR	mouse	3C6	Ventana	prediluted	IHC V
CK-19	rabbit	E16-L	DB Biotech	1/100	IHC
c-myc	mouse	9E10	Santa Cruz Biotechnology	1/100	IHC

Table S1: Description of the antibodies used

IF: immunofluorescence; IHC: immunohistochemistry; IHC V: IHC by Ventana; IP: immunoprecipitation; WB: Western blot
