# Supplementary material for: The Laminin Response in Inflammatory Bowel Disease: Protection or Malignancy?
Source: PLoS One. 2014 Oct 27;9(10):e111336. doi: 10.1371/journal.pone.0111336 (PMC4210184; doi:10.1371/journal.pone.0111336)
Supplement: Table S2 — Sequences of the primers used for genotyping, RT-qPCR and chromatin immunoprecipitation. (PDF) [file pone.0111336.s011.pdf]

Gene	Sequences (5' to 3')
Tg-*lama1*	GCCTCATTCCGGGGCTGTGTG
	AATGTGGTATGGCTGATTATG
Tg-*lama5*	CTGATCAATGGGGCCCCTGTCAAC
	AATGTGGTATGGCTGATTATG
LAMA1	GACAGCCCGGTGTCTGCCTTCACG
	GTGGCGGTTTTGGGCTCATATGCA
LAMA5	TCCCCTACTGCGAAGCTG
	CCTCAGGAAGGGCAGGAT
LAMA3	TGCCCATGTCCTCACACTAA
	CACGTCTCCCCCATTCAC
LAMB1	CCACTGAAAAACATTGGGAATC
	TGAGCCATCATTTCTGTAACATC
LAMC1	CACAGAGGCCAAGAACAAGG
	CTTGGTGCTGGTGGCATT
TP53	QuantiTect® primer assay Quiagen
TP53 site 1	TGGTGGGATGATAATGAGATATTGTCC
	CTGAGGCAGGAGAATTGCTTGAAC
TP53 site 2	GAATGAATGAAGTAGGCACCATTGC
	TCAGGTGGTCAGAAGATGGGTAGAG
TP53 site 3	ATCATTTGACATGTGCAAGGCAGAG
	CTGAGGCAGGAGAATGGCATGAA
TP53 site 4	AGATGTCCAAGCTGTACCCCATACC
	AGGCCTGAGTTCCTGCATTTCTAAC
TP53 site 5	CTATGTGGGTGTCAGGATACGAGGA
	TGAAGCAAAGTTTCAGAAACACAGC
TP53 site 6	TGATGCCAGGAACTACGCTAACAAT
	CCAGCTCTCACGTTTACTTGATTGG
TP53 intron	CCATCTCGATAACCGCTCCTACTCT
	AACTTATAGGGTGGGCCCTGGAGAA

Table S2: Sequences of the primers used for genotyping, RT-qPCR and chromatin immunoprecipitation
