# Supplementary material for: The Laminin Response in Inflammatory Bowel Disease: Protection or Malignancy?
Source: PLoS One. 2014 Oct 27;9(10):e111336. doi: 10.1371/journal.pone.0111336 (PMC4210184; doi:10.1371/journal.pone.0111336)
Supplement: Table S3 — List of the markers examined to characterize the UACL. (PDF) [file pone.0111336.s012.pdf]

| marker | function | positivity in UACL | colonic epithelial cells (outside UACL) |
|---|---|---|---|
| Pdx1 | transcription factor; role in decision of cell fate | Supplementary Figure 4 | negative (normal and IBD) |
| Sox9 | transcription factor; role in decision of cell fate | Supplementary Figure 4 | positive in crypts (normal and IBD) |
| β-catenin | adherens junction protein (membrane)<br>Wnt-stimulated effector (nuclear) | nuclear staining; Supplementary Figure 4 | membrane staining (normal and IBD) |
| E-cadherin | epithelial transmembrane glycoprotein | decrease of intercellular staining; Supplementary Figure 4 | membrane staining (normal and IBD) |
| CK-19 | epithelial cytoskeletal protein | moderate staining; Supplementary Figure 4 | membrane staining (normal and IBD) |
| p53 | tumor suppressor gene; cell cycle regulator | nuclear staining; Figure 1 | rare nuclear staining (normal)<br>scarce nuclear staining (IBD) |
| 53BP1 | tumor protein p53-binding protein1<br>player in the cellular response to DNA damage | not illustrated | localized to base of the gland (normal); positive (IBD) |
| Mlh1 | mismatch repair gene | Supplementary Figure 4 | weak and localized to base of the gland (normal); positive (IBD) |
| Msh2 | mismatch repair gene | Supplementary Figure 4 | weak and localized to base of the gland (normal); positive (IBD) |
| c-myc | oncogene; Wnt target gene; control of DNA replication | Supplementary Figure 4 | positive in crypts (normal and IBD) |
| MUC5AC | secreted mucin glycoprotein, expressed in the stomach; described either in reparation or tumorigenesis process in intestine | Supplementary Figure 3 * | negative (normal and IBD) |
| MUC6 | secreted mucin glycoprotein, expressed in stomach and Brunner's glands | Supplementary Figure 3 * | negative (normal and IBD) |
| TFF1 | protein of the trefoil factor family; expressed in stomach | Supplementary Figure 3 * | negative (normal); some positive (IBD) |
| **marker** | **function** | **absence in UACL** | **colonic epithelial cells (outside UACL)** |
| MUC2 | secreted mucin glycoprotein; expressed in the intestine | Supplementary Figure 3 | positive (normal and IBD) |
| TFF3 | protein of the trefoil factor family; expressed in the intestine | Supplementary Figure 3 ** | positive (normal and IBD) |
| Cdx2 | homeobox gene; specific of adult intestine; implicated in colorectal cancer | Supplementary Figure 3 | positive (normal and IBD) |
| bcl2 | anti-apoptotic role | not illustrated | some positive in crypts (normal and IBD) |
| Ki67 | proliferative marker | Supplementary Figure 4 | positive; base of the gland (normal and IBD) |
| TUNEL | apoptosis | Supplementary Figure 4 | rare desquamating epithelial cells (normal and IBD) |
| Mdm2 | regulator of p53 | not illustrated | rare (normal and IBD) |
| γH2AX | phosphorylated form of histone H2AX; indicator for DNA damage; recruitment of repair proteins | not illustrated | rare positive cells (normal and IBD) |
| EGFR | EGF receptor, activated in cancer | not illustrated | expression in colonocytes (normal); sporadic in IBD |

* Proportion of positive cells in UACL is variable according to location

** The loss is variable according to location of UACL

**Table S3: List of the markers examined to characterize the UACL**
