# Supplementary material for: The Laminin Response in Inflammatory Bowel Disease: Protection or Malignancy?
Source: PLoS One. 2014 Oct 27;9(10):e111336. doi: 10.1371/journal.pone.0111336 (PMC4210184; doi:10.1371/journal.pone.0111336)
Supplement: Methods S1 — Mouse models; Histology and immunodetection; Cell culture conditions; quantitative RT-PCR. (PDF) [file pone.0111336.s013.pdf]

Methods S1

Mouse models, genotyping and animal experiments

Plasmid constructs

The plasmid pBS-villin promoter containing 3.5 kb of the murine promoter, the first non coding exon, the 5.5 kb of the first intron and a small part of the second villin exon [1] was kindly provided by Sylvie Robine (Institut Curie, Paris, France). For the LMα5 plasmid construct, a double strand annealed oligonucleotide, 5'GGTTTAAACATTTAAATGGTA3' and 5' CATTTAAATGTTTAAACCGTA3', was inserted into the KpnI site of the vector pBS-villin promoter MES SV40 polyA (described in Mammadova-Bach et al., submitted), to generate the plasmid pBS-villin promoter MES KSP SV40 polyA which has now SwaI and PmeI unique sites. A double strand annealed oligonucleotide, 5'CGCGAGGCCGGCCACCGGTGGCGCGCCA3' and 5'CGCGTGGCGCGCCACCGGTGGCCGGCT3', was inserted in the MluI site of the vector pBS-villin promoter MES KSP SV40 polyA, generating the plasmid pBS-villin promoter MES KSP MAAF SV40 polyA which has now AscI, AgeI and FseI unique sites. A double strand annealed oligonucleotide, 5'GGCCGCATTTAAATGTTTAAACCC3' and 5'GGCCGGGTTTAAACATTTAAATGC3', was inserted in the NotI site of the vector pBS-villin promoter MES KSP MAAF SV40 polyA, generating the plasmid pBS-villin promoter MES KSP MAAF NSP SV40 polyA which has now two SwaI and PmeI sites flanking the villin promoter and the SV40 sequence.

The plasmid pGEM mTieI *lama5* containing the mouse full length cDNA encoding for the *lama5* gene was generated by Dr L. Sorokin (Institute of Physiological Chemistry and Pathobiochemistry, University of Münster, Germany). The unique EcoRI site presents in this

vector was destroyed using the T4 polymerase. Site directed mutagenesis was then used to insert a BsiWI site (5'phosphorylated CCCCGGGCCCACTAGCGTACGATGGCGAAGCGCGGA3') before the initiation codon, an EcoRI site (5'phosphorylated GCGCACTCCCGGGGGAATTCCTTCAGCCTGCACCC3') just after the signal peptide sequence and an AscI site (5'phosphorylated CCGCTACTTTGGCATGGCGCGCCTTAATTAAGTGACAT3') just before the stop codon of the mouse *lama5* cDNA. A double strand annealed oligonucleotide, 5'AATTCGGGTACCGACTACAAAGACGATGACGACAAA3' and 5'AATTTTTGTCGTCATCGTCTTTGTAGTCGGTACCCG3', containing a Flag tag sequence was then inserted into the EcoRI site. A double strand annealed oligonucleotide, 5'CGCGGGATATCCCTATGACGTCCCAGACTATGCCTAAGG3' and 5'CGCGCCTTAGGCATAGTCTGGGACGTCATAGGGATATCC3' containing a HA tag sequence and a stop codon was inserted into the AscI site. The BsiwI-AscI fragment containing the *lama5* cDNA was purified and inserted into the BsiwI-AscI sites of the pBS-villin promoter MES KSP MAAF NSP SV40 polyA vector generating the pBS-villin promoter *lama5* tag construct.

The functionality of the pBS-villin promoter *lama5* tag was tested by transient transfection in the HEK293 cell line. 75x 10^3 cells/well in Lab-Tek™ (Nunc, France) were transfected with 1µg of pBS-villin promoter *lama5* tag using JetPEI reagent (PolyPlus Transfection, Illkirch, France) according to the manufacturer's instructions. At 24h after transfection, cells were fixed with 3% paraformaldehyde (10mn) and then treated with 0.5% Triton X-100 (10mn) in PBS. After washing 3 times with PBS, transfected cells were incubated for 1 h at room temperature with rabbit polyclonal anti-HA antibodies diluted to 1/50 (H6908, Sigma-Aldrich, France) or mouse monoclonal anti-Flag antibodies diluted to 1/1000 (M2, Sigma-

Aldrich, France). After washing in PBS (3 times) cells were incubated 1h with the secondary antibodies. Cells were examined using a Leica DMR 2 fluorescent microscope.

The same procedure was used to generate the plasmid pBS-villin promoter *lama1* (described in Mammadova-Bach et al., submitted) using the full length cDNA encoding the mouse *lama1* gene (kindly provided by Pr. Peter Yurchenco, Robert Wood Johnson Medical School, Piscataway, NJ 08854, USA).

Tg-lama1 and Tg-lama5 mice

Transgenic mice overexpressing the LMα1 or the LMα5 chain in their intestinal epithelial cells under the control of the villin promoter were generated. Generation of Tg-*lama1* mice was described in Mammadova-Bach et al. (submitted). Concerning the Tg-*lama5* mice, the SwaI fragment containing the villin promoter following by the mouse *lama5* cDNA and the SV40 polyA from the pBS-villin promoter *lama5* plasmid was purified and used to inject pronuclei of fertilized oocytes prepared from the hybrid line B6D2 (C57Bl/6xDBA/2) at the transgenic facility of the IGBMC (Illkirch, France). Germline transmission was detected by polymerase chain reaction analysis of tail DNA obtained at weaning, using the villin2 primer (5' GGCAGGGTAGAGGTTGGACTA3') and the primer (5'TCGTCATCGTCTTTGTAGTCG3') located in the Flag tag generating a fragment of 300 bp. This was confirmed using the primers Lna5s4 present in the 3' region of the *lama5* cDNA (5'CTGATCAATGGGGCCCCTGTCAAC3') and the SV40 3' down generating a fragment of 240 bp. 300 oocytes were injected giving raise to 53 living mice. 13 founder lines have integrated the transgene and two were kept because of their high expression of LMα5.

The various transgenic lines studied developed and bred normally without any obvious gastrointestinal defects. The expression of the transgenes in the gut of adult mice was checked

by Northern blot, RT-qPCR or immunodetection. All procedures with animals were performed according to INSERM ethical criteria.

Induction of colitis and of cancer-associated colitis

Colitis was induced by providing animals with drinking water containing dextran sulphate sodium (DSS) 3% (wt/vol) for 6 days; mice were then sacrified 3 or 5 days after as described previously [2]. Inflammation was scored according to the degree and extent of inflammation: overall number of inflammatory cells (scale: 0-3), epithelial damage (scale: 0-3), percentage of mucosal injury: grade 1 (1-25%), grade 2 (26-50%), 3 (51-75%), 4 (76-100%) [3]. In total, the severity of the colitis was graded on a scale 1-10. In parallel, concentrations of inflammatory cytokines (TNF-α, IL-1β, IL-6 and IL-12) in distal colon were measured using a Cytokine Enzyme-Linked Immunosorbent Assay (ELISA, BD Biosciences) as previously described [4] (n=6, in duplicate).

To induce colorectal carcinogenesis associated with chronic colitis two protocols were used (**Figure 4A**). In the first model, mice were subjected to a combined treatment of AOM (azoxymethane) and DSS [5]. Briefly, mice were injected intraperitoneally with 10 mg/kg body weight of AOM dissolved in PBS. On the following day, cyclic administration of DSS was started, with each cycle consisting of one week of 3% DSS and two weeks of water. Mice received two cycles of DSS and were sacrificed 3 days after the last day of DSS treatment. In the second model, mice were given 3 cycles of DSS: 3% DSS for 1 week followed by regular water for the next 2 weeks. The mice were sacrificed at week 38. The entire colon and rectum was removed, opened longitudinally, and rolled with the mucosa inwards, the so-called Swiss-roll method. Histological examination and immunodection were performed on frozen sections.

Immunohistochemistry, immunofluorescence

For immunohistochemistry, tissue sections of 6μm thickness were used. For some antibodies, tissue sections were treated with a Ventana Benchmark® automaton (Ventana Medical Systems). For other antibodies, tissue sections were deparaffinized with toluene, then boiled with the antigen retrieval sodium citrate buffer (pH6) for 10 min. Sections were incubated overnight at 4°C with primary antibodies or for 20-30 min at 42°C for sections processed by Ventana (**Table S1**). Slides were then incubated with biotinylated secondary antibodies (Vector Laboratories), amplified with the ABC Elite Vectastain kit and developed with the DAB kit from Vector Laboratories. For immunofluorescence, unfixed human or mouse intestinal frozen sections of 5μm were incubated overnight directly with the primary antibody. Detection of intracellular LMα1 chain present as individual dots scattered within the cell, was performed on untreated and irinotecan-treated (20μM, 48h; MYLAN, Saint Priest, France) HCT116 cells permeabilized with 0.1% Triton X-100 for 10 min using LMα1 G4/G5 antibodies. Semi-quantitative determination of individual fluorescent points was performed by using the ImageJ processing program. Bound antibodies were visualized with anti-mouse, anti-rabbit or anti-rat secondary antibody conjugated with Alexa 488 (Molecular Probe) or Cy3 (Jackson ImmunoResearch, UK) or anti-mouse IgM conjugated with FITC (Sigma, Lyon France). DAPI was used to visualize nuclei. After mounting in a glycerol/PBS/phenylenediamine solution, sections were examined using an epifluorescence microscope (AX 60, Olympus Optical Co, Hamburg, Germany). Pictures were taken with an Olympus digital camera. Control sections were processed as above with omission of the primary antibodies.

Histology, assessment of apoptosis

For histological examination and immunohistochemical analyses, intestines were prepared by the Swiss-roll method. Samples were either directly fixed in 4% paraformaldehyde or embedded in Tissue-Tek. Sections of 7 µm were prepared for standard hematoxylin/eosin staining (HE) or periodic acid Schiff (PAS). For detection of apoptosis, the ApopTag® Plus Peroxidase *In Situ* Apoptosis Detection Kit (Millipore, France) was used according to the manufacturer's guide.

Cell culture conditions

HCT116 human colon carcinoma cells were maintained in culture at 37°C in DMEM medium supplemented with 10% fetal calf serum and gentamycin (Invitrogen, France). For Western blot analysis, proteins were extracted from HCT116 cells that have been plated on LM substrata, in a 50mM Tris-HCl pH8, 1% NP40 solution with protease and phosphatase inhibitors (Roche Applied Science). The proteins were separated by SDS-PAGE analysis (10% gels) and subjected to Western blotting. HT-29/κB-Luc-E cells [6] were cultured in RPMI 1640 medium with glutamine, 10% heat-inactivated fetal calf serum, peni-streptomycin (Invitrogen, France), hygromycin (200µg/ml; Invitrogen, France) and with human TNF-α (10ng/ml; Preprotech).

RNA extraction and quantitative RT-PCR

RNA was extracted using TRI Reagent (Molecular Research Center Inc., Euromedex, France) and further purified by an additional phenol/chloroform 5/1 (V/V) extraction. The amounts of RNA were measured using a Nanodrop (ThermoScientific NanoDrop 1000). Total RNA was treated with DNase I (Roche Diagnostics, Meylan, France). cDNA synthesis was performed using the High Capacity cDNA RT kit (Applied Biosystems Inc, France). For quantitative real-time PCR (qPCR) analysis, we used the LightCycler™ system (Roche Diagnostics,

Meylan, France) or the 7500 real-time PCR system (Applied Biosystems Inc, France). PCR amplification was performed using the FastStart DNA Master Mix SYBR Green I (Roche Diagnostics, Meylan, France) or using the TaqMan® Gene Expression Master mix for *GAPDH* or the Power SYBR® Green PCR Master Mix (Applied Biosystems Inc, France). PCR efficiency (E) determined by serial dilution of a pool of cDNA was calculated by the slope of the regression line ($E = 10^{-1/\mathrm{slope}} - 1$) and was higher than 80%. The specificity of the PCR products was assessed by generating a melting curve. The comparative CT method (ΔΔCT method) was used to quantify the cDNA of interest relative to the *GAPDH* reference. Data are represented as ratios of mean values (+/- SEM).

References

1. Pinto D, Robine S, Jaisser F, El Marjou F, Louvard D (1999) Regulatory sequences of the mouse villin gene that efficiently drive transgenic expression in immature and differentiated epithelial cells of small and large intestines. J Biol Chem 274: 6476-6482.

2. Wirtz S, Neufert C, Weigmann B, Neurath MF (2007) Chemically induced mouse models of intestinal inflammation. Nat Protoc 2: 541-546.

3. Dieleman LA, Palmen MJ, Akol H, Bloemena E, Pena AS et al. (1998) Chronic experimental colitis induced by dextran sulphate sodium (DSS) is characterized by Th1 and Th2 cytokines. Clin Exp Immunol 114: 385-391.

4. Barreau F, Madre C, Meinzer U, Berrebi D, Dussaillant M et al. (2010) Nod2 regulates the host response towards microflora by modulating T cell function and epithelial permeability in mouse Peyer's patches. Gut 59: 207-217.

5. Neufert C, Becker C, Neurath MF (2007) An inducible mouse model of colon carcinogenesis for the analysis of sporadic and inflammation-driven tumor progression. Nat Protoc 2: 1998-2004.

6. Kaci G, Lakhdari O, Dore J, Ehrlich SD, Renault P et al. (2011) Inhibition of the NF-kappaB pathway in human intestinal epithelial cells by commensal Streptococcus salivarius. Appl Environ Microbiol 77: 4681-4684.
